# The Effect of Resistance and/or Aerobic Training on Quality of Life, Fitness, and Body Composition in Prostate Cancer Patients—A Systematic Review and Meta-Analysis

**DOI:** 10.3390/cancers16244286

**Published:** 2024-12-23

**Authors:** Shimon Kempin, Alexander Buchner, Sarah Frederike Brose, Nina Schmidt-Hegemann, Matthias May, Ingmar Wolff, Anton Kravchuk, Christian Stief, Sabine D. Brookman-May, Benazir Enzinger

**Affiliations:** 1 Department of Urology, LMU University Hospital, Ludwig Maximilian University of Munich, 81377 Munich, Germany; 2 Department of Radiation Oncology, LMU University Hospital, Ludwig Maximilian University of Munich, 81377 Munich, Germany; 3 Department of Urology, St. Elisabeth Hospital Straubing, Brothers of Mercy Hospital, 94315 Straubing, Germany; 4 Department of Urology, University Medicine Greifswald, 17489 Greifswald, Germany; 5 Aura Biosciences, Boston, MA 02115, USA

**Keywords:** prostate cancer, physical activity, exercise intervention, muscle strength, cardiovascular health, fatigue management, functional capacity, mental health, side effects of androgen deprivation therapy, physical rehabilitation, biomarkers, fitness tests, depression reduction, strength training, endurance training

## Abstract

**Background**: Prostate cancer (PC) and its treatment are often associated with side effects such as fatigue, muscle loss, and diminished quality of life (QoL). Physical exercise, particularly resistance training (RT) and aerobic training (AT), has been suggested as a strategy to mitigate these effects. However, the comparative efficacy of RT, AT, and combined RT/AT on QoL, body composition, physical fitness, and laboratory markers in PC patients is still insufficiently understood. **Methods**: Randomized controlled trials (RCTs) investigating structured RT, AT, or combined RT/AT programs in PC patients undergoing various treatments were included. The primary outcome was QoL, assessed using EORTC QLQ-C30 and EORTC QLQ-PR25 questionnaires. Secondary outcomes included body composition, fitness, and laboratory parameters. The studies were sourced from PubMed, Embase, and CENTRAL through May 2024. The effect sizes were pooled using random-effects models, and the risk of bias was systematically assessed following the GRADE approach. **Results**: A total of 30 RCTs, encompassing 2216 PC patients, were analyzed. Combined RT/AT significantly improved QoL subdomains, including global health, and cognitive and sexual function, while reducing fatigue and urinary symptoms. RT alone improved body composition by increasing lean body mass and reducing body fat percentage. Both RT and combined RT/AT enhanced strength (chest and leg press) and VO_2_peak. No significant changes were observed in laboratory markers, such as PSA or lipid levels. The effects of isolated AT remain unclear due to limited data. **Conclusions**: RT and combined RT/AT significantly improve QoL, fitness, and body composition in PC patients, with no detectable effect on PSA or lipid levels. Further research is needed to elucidate the specific effects of AT and to investigate long-term outcomes.

## Simple Summary

Worldwide, prostate cancer is the second most common cancer affecting men. Both the disease and treatments like surgery, radiotherapy, or drug therapy can cause unpleasant side effects, such as fatigue, muscle loss, and reduced quality of life. Exercise, particularly strength training and aerobic workouts, has been suggested to help reduce these effects. This study reviewed findings from controlled trials to evaluate how strength training, aerobic exercise, or a combination of both, affected men receiving prostate cancer treatment. The results show that combining strength and aerobic exercise significantly improves quality of life, especially in general health, mental clarity, and sexual wellbeing. Strength training alone helped by increasing muscle mass and lowering body fat. Both types of exercise improved physical strength and fitness. However, the training had no impact on laboratory results like PSA or cholesterol levels. More research is needed on long-term effects, particularly of aerobic exercise alone.

## 1. Introduction

Prostate cancer (PC) is the second most prevalent cancer in men, with approximately 1.4 million new cases reported worldwide in 2020 [1]. Among men aged 60 years and older, PC is the most common type of cancer [1]. Standard of care (SOC) treatments for PC, depending on the disease stage, include radical prostatectomy (RP), external beam radiotherapy (EBRT), and systemic therapies, alone or in combination.

While localized PC rarely causes direct symptoms, adverse effects of RP and EBRT are common, including urinary incontinence, erectile dysfunction, and bowel dysfunction [2]. For locally advanced and metastatic PC, disease- or treatment-related symptoms include urinary retention, hydronephrosis, anemia, fatigue, erectile dysfunction, metastatic bone pain, pathologic fractures, and weight loss. Androgen deprivation therapy (ADT), a form of chemical castration, particularly increases fatigue and fat mass, decreases lean body mass (LBM), bone mineral density, and strength levels, and negatively impacts sexual function and quality of life (QoL) [3,4,5]. ADT also raises the risk of obesity, diabetes, cardiovascular disease, and depression [6,7,8]. Additionally, androgen receptor signaling inhibitors (ARSI), such as apalutamide, enzalutamide, and darolutamide, can cause fatigue, impair cognitive and physical function, and increase the risk of bone fractures and falls [9]. When chemotherapy is combined with ADT, additional adverse effects, such as nausea, malaise, neutropenia, and neuropathy, are more common [10].

Considering the increasing life expectancy and rising PC incidence due to improved screening and diagnostic abilities, efforts in improving QoL as well as physical health in the older PC population became of vital importance. The increase in healthy life expectancy comes from the declining mortality rate rather than reduced years lived with disability. In the past decade, physical exercise, particularly resistance training (RT) and aerobic training (AT), has been increasingly recognized as a valuable tool to mitigate the side effects of PC treatment and counteract disease-related impairments. Several studies involving men on ADT have shown that RT and/or AT can improve muscle strength and physical performance [11,12,13,14], alleviate psychiatric stress [15], and help maintain sexual function [16]. A study by Kang et al. (2021) found that high-intensity interval training (HIIT) improved cardiorespiratory fitness, reduced levels of prostate-specific antigen (PSA), and inhibited PC cell growth in men under active surveillance (AS) [17]. RT and AT have also been found to reduce adverse effects of ADT on body composition [18,19]. Furthermore, Kim et al. (2022) demonstrated that combined RT and AT reduced cell proliferation rates in men with metastatic castration-resistant PC (mCRPC), suggesting an oncological benefit [20].

Previous meta-analyses consistently reported positive effects of exercise on body composition, lower and upper body strength, and aerobic fitness [21,22,23,24,25,26]. The impact of exercise on QoL in the PC population is a relatively new research area. A meta-analysis by Yang et al. (2017) demonstrated improvements in cancer-related fatigue and QoL with physical exercise [27], while other studies found no effect on QoL [21] or fatigue [25]. A recent trial investigating the effects of RT and AT on QoL in mCRPC patients found little to no improvements and even some negative effects on certain QLQ-C30 subscales, including sleep quality [28]. Most of the previous studies focused on patients receiving ADT and implemented mixed RT/AT or RT-only interventions [22,25,26,27]. Lopez et al. (2021) conducted univariate meta-regressions and dose–response analyses to explore whether outcome variations could be attributed to exercise type, intensity, weekly volume, or intervention duration [24]. They found that some fitness parameters, such as seated row strength gains, were more pronounced in RT-only interventions compared to the mixed RT/AT interventions. However, AT’s influence on these parameters was not addressed. Currently, the effects of isolated AT on anthropometrics, fitness levels, and QoL in PC patients remain unclear, and more data are needed for patients undergoing treatments other than ADT.

In this systematic review and meta-analysis, we investigate the effects of RT, AT, and combined RT/AT on QoL, body composition, fitness, laboratory health parameters, and PSA levels in a population of PC patients.

## 2. Methods

### 2.1. Study Selection Procedure

This study was conducted in accordance with the PRISMA guidelines [29]. The review was registered in the PROSPERO database under the ID CRD42024552379. No study protocol was published prior to the performance of this review.

The research question was structured using the PICOS (Population, Intervention, Comparator, Outcome, Study design) framework. The detailed PICOS design is shown in Table S1. The Electronic databases PubMed, Embase, and Cochrane Central Register of Controlled Trials (CENTRAL) were systematically explored up to May 2024. The search strategy “(randomized controlled trial) AND (prostate cancer OR prostatic neoplasms) AND (physical exercise) NOT (pelvic floor exercise)” was used to find relevant trials.

Studies were included if they were randomized controlled trials (RCTs) conducted on PC patients aged > 18 years at any disease stage (i.e., localized, locally advanced, or advanced PC), who received active therapy (RP, EBRT, or systemic treatment). Patients had to be provided with a structured RT and/or AT program that could be performed at home or at a centralized facility with or without supervision. The primary outcome of this review was QoL via the EORTC QLQ-C30 [30] and EORTC QLQ-PR25 [31] questionnaires. Secondary outcomes were body composition (body weight, body mass index (BMI), LBM, body fat percentage), laboratory markers (blood lipids, PSA), and fitness parameters (chest press/leg press strength, VO_2_peak).

Studies were excluded if (1) the training program included only pelvic floor exercises; (2) the study did not report on any of the mentioned outcomes; (3) the study included a mixed cancer cohort and did not report separate outcomes of PC patients only; (4) the study did not implement a standard of care (SOC) or usual care (UC) control group; (5) the participants did not receive an active therapy in the 12 months prior to the intervention; (6) pharmacologically active agents (e.g., metformin) were used in addition to the intervention; (7) the results were published in a language other than English.

After extraction of all search results into an Excel sheet, duplicates were removed before proceeding to title and abstract screening. The latter was performed by B.E. and S.K., with the studies sorted by database and the work divided between the two reviewers. Abstracts that did not provide conclusive information about the in-/exclusion criteria were selected for full-text evaluation. Subsequently, these full texts were screened for their relevance according to the pre-defined eligibility criteria. Any disagreements between reviewers were resolved by consensus.

### 2.2. Data Extraction

Data were extracted from the remaining studies via a standardized Excel form. Data extraction was performed by B.E., S.K., and S.F.B., with the workload being divided between the reviewers. The following items were extracted:

(a) Study characteristics: year of publication, first author, country/countries, number of study sites, number of patients in total and per group, type of intervention (RT, AT, or combination), duration of intervention, training frequency (sessions per week), degree of personal guidance (supervised, unsupervised, or combination).
(b) Patient characteristics: mean age in total and per group, disease stage (localized, locally advanced, or advanced PC), current therapy (RP, EBRT, ADT, ARSI, chemotherapy, or combination).
(c) Outcomes:
  - QoL: QLQ-C30 subcategories (global health, physical functioning, emotional functioning, cognitive functioning, fatigue, pain), QLQ-PR25 subcategories (sexual activity, sexual functioning, urinary symptoms, bowel symptoms, hormonal treatment symptoms).
  - Body composition: bodyweight (kg), BMI (kg/m^2^), LBM (kg), body fat percentage (%).
  - Laboratory parameters: total cholesterol (mmol/L), triglycerides (TAG; mmol/L), low-density lipoprotein (LDL; mmol/L), high-density lipoprotein (HDL; mmol/L), PSA (ng/mL).
  - Fitness parameters: one-repetition maximum (RM) chest press (kg), 1-RM leg press (kg), VO_2_peak (mL/kg/min).

These outcome parameters were selected for this meta-analysis because they were the most common ones measured in the selected studies.

For all continuous outcomes, the mean with standard deviation (SD) for each group before and after the intervention was extracted. The intervention effect (IE), defined as the group difference between the (adjusted) mean change (post-intervention–pre-intervention) between the intervention group and the control group, was extracted with its confidence interval (CI). In cases in which the IE was not reported in the publication, it was calculated manually.

Strength test data from studies that reported 3-RM instead of 1-RM, bench press instead of chest press, and leg extensions instead of leg press were extracted into the above-mentioned categories to avoid too many variables. Because of the numerical and physiological similarity of these outcomes, comparability can be assumed.

### 2.3. Statistical Analysis

For the meta-analysis, the interventions were separated into three groups: combined RT and AT, RT only, and AT only. The pooled-effect estimates were obtained from the IE for each intervention group. Calculations were performed using a random-effects model. The level of significance was set at *p* ≤ 0.05. Statistical heterogeneity was assessed using Cochran’s Q test, with *p* ≤ 0.10 indicating significant heterogeneity. The I^2^ (inconsistency) statistic was calculated and interpreted as follows: I^2^ < 30% = low heterogeneity, 30% ≤ I^2^ ≤ 60% = moderate heterogeneity, I^2^ > 60% = high heterogeneity. All calculations were performed using the software MedCalc version 23 (MedCalc, Ostend, Belgium).

### 2.4. Risk of Bias and Certainty of Evidence Assessment

Two authors (S.K., S.B.) independently evaluated the risk of bias (RoB) in the included studies. Specifically, the domains “randomization process”, “blinding”, “incomplete outcome data”, and “selective reporting” were systematically assessed. The overall RoB for each outcome was categorized as “low”, “some concerns”, or “high”. Any discrepancies were resolved through discussion or consultation with another author (B.E.). The presence of publication bias was explored using contour-enhanced funnel plots along with Egger’s test and Begg’s test, considering *p* ≤ 0.05 as indicative of publication bias. The GRADE approach was employed to assess the certainty of evidence for each outcome, conducted by authors B.E. and S.K. [32,33].

## 3. Results

### 3.1. Studies Included

**Figure 1:**
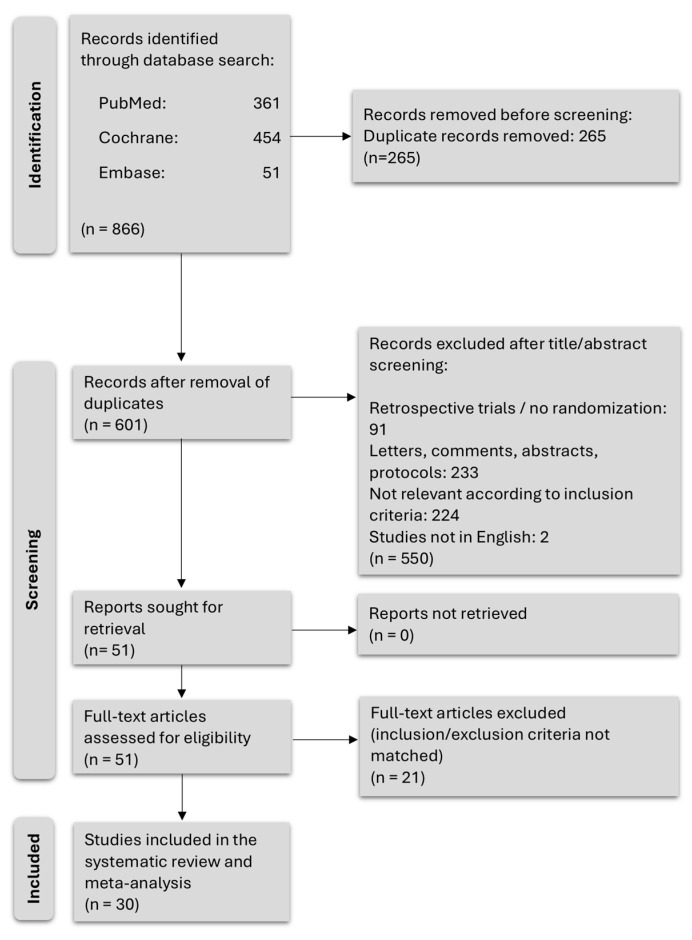
Flow diagram of study selection process.

The initial search strategy retrieved 866 results, with 601 studies remaining after removal of duplicates. In total, 550 studies were excluded by title and abstract screening and 51 full texts were assessed for eligibility. Of these, 30 studies fulfilled our inclusion and exclusion criteria and therefore were included in the present review and meta-analyses. Since eight studies included not one but two intervention groups, a total of 38 intervention groups could be defined and statistically compared to the SOC. A flow diagram showing the study selection process can be found in Figure 1.

Some studies first appeared to meet the eligibility criteria but were excluded later. One example is a trial by Hackshaw-McGeagh et al. (2019), in which the exercise intervention consisted of brisk walking for 30 min on at least 5 days a week [34]. This did not meet our criterium of structured aerobic training and, thus, the study was excluded upon closer examination. Further, several studies that initially were deemed eligible were later excluded because the reported outcomes did not overlap with those selected for this review.

### 3.2. Patient and Intervention Characteristics

The study characteristics are displayed in Table 1. A total of 2216 PC patients with an average age of 69.1 ± 2.5 years participated in the included studies. Eight studies included only patients with advanced PC, 12 studies included only patients with localized or locally advanced PC, five studies reported the outcomes of a mixed cohort of different disease stages, and five studies did not specify the disease stage of their cohort. The most common therapy regime used was systemic therapy, with 21 studies including patients under ADT/ARSI/chemotherapy. EBRT (with or without additional RP or systemic therapy) was the second most used therapeutic option, appearing in 17 studies. RP (with or without EBRT or systemic therapy) was the least common treatment option, applied in five studies.

The study by Ax et al. (2022) implemented a mixed cancer cohort (breast, colorectal, and prostate cancer) [35]. Because of the separately reported QoL outcomes, it was included in this review.

In total, 18 studies reported data on body composition [11,12,20,36,37,38,39,40,41,42,43,44,45,46,47,48,49,50], 10 studies on laboratory parameters [11,38,39,40,41,45,47,49,51,52], 12 on QoL [11,16,28,35,38,40,44,53,54,55,56,57], and 20 on fitness parameters [11,12,15,36,37,39,40,42,43,44,45,46,47,48,49,51,52,55,56,58]. In one study, an average strength composite of four exercises, including the leg press, was reported [15]. To avoid creating too many strength subcategories, we included these data in the 1-RM leg press category.

Of the 38 intervention groups, 13 implemented RT only [15,28,42,43,44,45,46,47,48,49,51,52], 5 AT only [28,48,49,50,52], and 20 a combination of RT and AT [11,12,15,16,20,35,36,37,38,39,40,41,42,53,54,55,56,57,58]. The mean intervention duration was 20.6 ± 11.3 weeks, ranging from 8 to 52 weeks (1 year). The studies implemented a mean of 2.9 ± 0.8 training sessions per week. Some studies examined the effect of an exercise intervention together with some additional variables. RT in the study by Galvão et al. (2021) also included impact loading (in the form of jumps) [15]. Another study additionally investigated the effect of protein supplementation, with one RT intervention group receiving a protein supplement and the other a placebo, while the control group received UC without protein [43]. One other study added calcium, vitamin D and protein supplementation to the RT intervention [47], and in the study by Newton et al. (2020), the participants in both groups received vitamin D and calcium [58]. All these supplementary measures did not have a significant impact on the results of the present meta-analysis.

### 3.3. Effect of RT and/or AT on QoL

**Figure 2:**
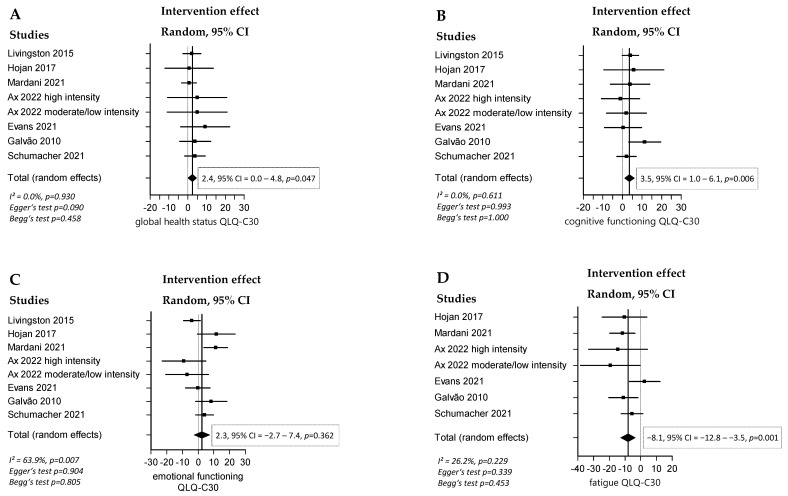

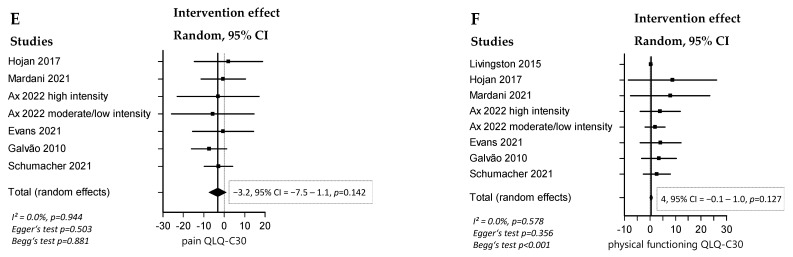
Forest plots with the intervention effects of combined RT and AT compared with SOC on the QLQ-C30 subcategories. Overall analysis conducted with a random-effects model. I^2^ represents the heterogeneity test. (**A**) Effect of combined RT and AT compared with SOC on QLQ-C30 subcategory global health status [11,35,38,53,54,55,56], (**B**) Effect of combined RT and AT compared with SOC on QLQ-C30 subcategory cognitive functioning [11,35,38,53,54,55,56], (**C**) Effect of combined RT and AT compared with SOC on QLQ-C30 subcategory emotional functioning [11,35,38,53,54,55,56], (**D**) Effect of combined RT and AT compared with SOC on QLQ-C30 subcategory fatigue [11,35,38,54,55,56], (**E**) Effect of combined RT and AT compared with SOC on QLQ-C30 subcategory pain [11,35,38,54,55,56], (**F**) Effect of combined RT and AT compared with SOC on QLQ-C30 subcategory physical functioning [11,35,38,53,54,55,56].

**Figure 3:**
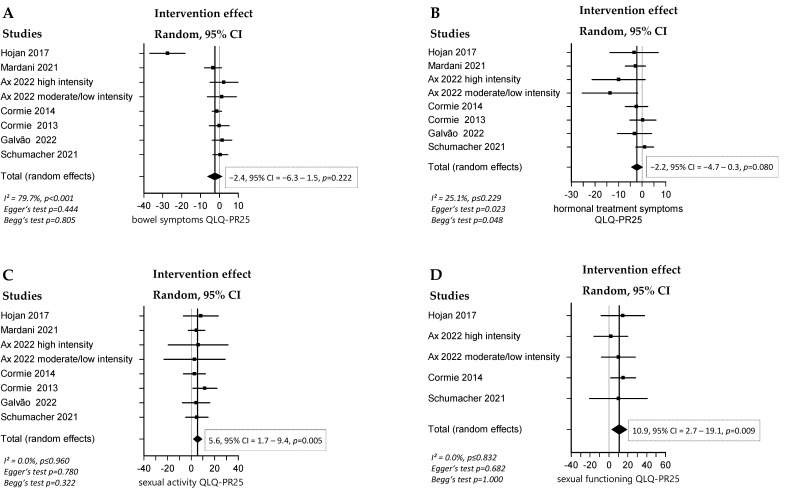

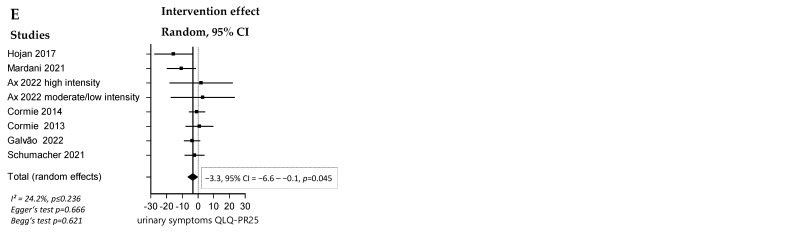
Forest plots with the intervention effects of combined RT and AT compared with SOC on the QLQ-PR25 subcategories. Overall analysis was conducted with a random-effects model. I^2^ represents the heterogeneity test. (**A**) Effect of combined RT and AT compared with SOC on QLQ-PR25 subcategory bowel symptoms [35,38,40,46,54,56,57], (**B**) Effect of combined RT and AT compared with SOC on QLQ-PR25 subcategory hormonal treatment symptoms [35,38,40,46,54,56,57], (**C**) Effect of combined RT and AT compared with SOC on QLQ-PR25 subcategory sexual activity [35,38,40,46,54,56,57], (**D**) Effect of combined RT and AT compared with SOC on QLQ-PR25 subcategory sexual functioning [35,38,40,56], (**E**) Effect of combined RT and AT compared with SOC on QLQ-PR25 subcategory urinary symptoms [35,38,40,46,54,56,57].

A significant positive overall effect of combined RT and AT on QoL, measured by the QLQ-C30 global health status (effect = 2.4, 95% CI = 0.0 to 4.8, *p* = 0.047), was found (Figure 2 and Figure 3). The intervention groups also scored better in QLQ-C30 subcategories such as cognitive functioning (effect = 3.5, 95% CI = 1.0 to 6.1, *p* = 0.006) and fatigue (effect = −8.1, 95% CI = −12.8 to −3.5, *p* = 0.001), as well as in the QLQ-PR25 subcategories sexual activity (effect = 5.6, 95% CI = 1.7 to 9.4, *p* = 0.005), sexual functioning (effect = 10.9, 95% CI = 2.7 to 19.1, *p* = 0.009), and urinary symptoms (effect = −3.3, 95% CI = −6.6 to −0.1, *p* = 0.045). No significant overall effect was detected in the QLQ-C30 subcategories emotional functioning, pain, and physical functioning, nor in the QLQ-PR25 subcategories bowel symptoms and hormonal treatment symptoms.

Due to the lack of studies examining solely the impact of RT or AT, no meta-analyses could be conducted examining the separate roles of RT or AT in QoL in PC patients. In two studies implementing isolated RT interventions, no significant effects could be detected in any QLQ-C30 subcategories [28,44]. Only one study implementing isolated AT reported on QoL, also with no significant effects in any QLQ-C30 subcategories [28].

### 3.4. Effect of RT and/or AT on Body Composition

Combined RT and AT showed a positive overall effect on body fat percentage (effect = −1.1%, 95% CI = −1.4 to −0.6%, *p* < 0.001) and *LBM* (effect = 0.6 kg, 95% CI = 0.2 to 0.9 kg, *p* = 0.001). However, no significant overall effects could be detected in *BMI* or body weight.

The examination of the overall effect of isolated RT on body composition showed consistent results with the above—decrease in body fat percentage (effect = −0.6%, 95% CI = −1.2 to −0.3%, *p* = 0.003) and increase in LBM (effect = 0.6 kg, 95% CI = 0.2 to 1.0 kg, *p* = 0.007). BMI and body weight were not significantly affected.

Since only two studies implementing isolated AT reported on the same body composition parameters [48,49,50], no meta-analysis could be conducted. Descriptively, neither of these studies reported a significant effect on body weight, BMI, LBM, or body fat percentage.

### 3.5. Effect of RT and/or AT on Fitness Parameters

Combined RT and AT significantly improved all the fitness parameters studied: *chest press* (effect = 4.3 kg, 95% CI = 3.4 to 5.3 kg, *p* < 0.001), leg press (effect = 20.2 kg, 95% CI = 13.7 to 26.8 kg, *p* < 0.001), and VO_2_peak (effect = 1.3 mL/kg/min, 95% CI = 0.6 to 2.0 mL/kg/min, *p* < 0.001).

Similarly, isolated RT also resulted in significant gains in chest press (effect = 6.0 kg, 95% CI = 2.4 to 9.6 kg, *p* = 0.001), leg press (effect = 18.6 kg, 95% CI = 8.7 to 28.6 kg, *p* < 0.001), and VO_2_peak (effect = 2.3 mL/kg/min, 95% CI = 0.8 to 3.8 mL/kg/min, *p* = 0.003).

Again, due to the lack of studies implementing isolated AT, a meta-analysis could not be performed. However, none of the analyzed studies reported a significant effect on the above-mentioned fitness parameters [48,49,52].

### 3.6. Effect of RT and/or AT on Laboratory Parameters

**Figure 4:**
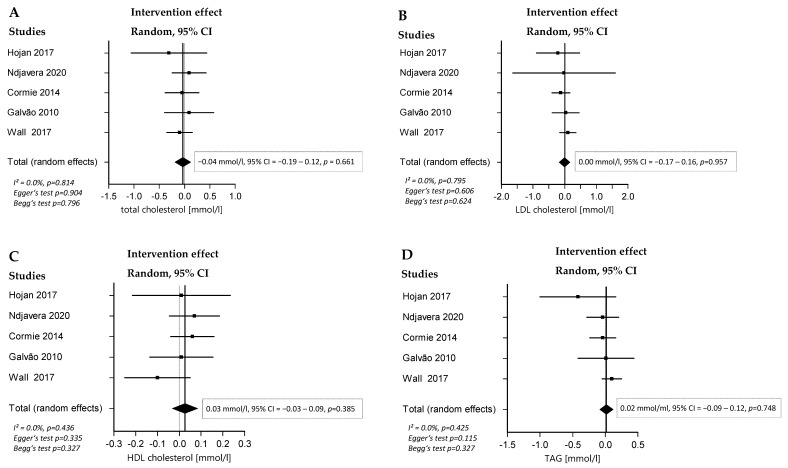
Forest plots with the intervention effects of combined RT and AT compared with SOC on total cholesterol [11,38,39,40,41] (**A**), LDL [11,38,39,40,41] (**B**), HDL [11,38,39,40,41] (**C**), and TAG [11,38,39,40,41] (**D**). Overall analysis was conducted with a random-effects model. I^2^ represents the heterogeneity test.

No significant overall effects of combined RT and AT or isolated RT could be detected in the lipid parameters (total cholesterol, LDL, HDL, and triglycerides). None of the studies implementing isolated AT reported on blood lipids. In regard to PSA kinetics, no significant findings were described in any of the studied groups (Figure 4).

### 3.7. Heterogeneity, RoB, and Certainty of Evidence Assessment

In the analysis of the primary endpoints, high heterogeneity (I^2^ > 60%) was observed only in the following subcategories: QLQ-C30 emotional functioning in the combined RT and AT group (I^2^ = 63.9%, *p* = 0.007), and QLQ-PR25 bowel symptoms in the combined RT and AT group (I^2^ = 79.7%, *p* < 0.001). High between-studies heterogeneity, ranging from I^2^ = 70.6% to 78.5%, was also detected in fitness parameters such as leg and chest press. A possible explanation for this may be the different reporting and measurement systems. As mentioned above, some studies published results for 3-RM instead of 1-RM, bench press instead of chest press, and leg extensions instead of leg press.

Possible publication bias was observed in the analysis of the effect of combined RT and AT on QLQ-C30 physical functioning (Begg’s test *p* < 0.001). One possible explanation for this could be that negative studies that did not achieve promised results in the boosting of the physical well-being of PC patients were not published to the same extent as positive studies.

The results of the RoB assessment are shown in Table S2. Participant blinding is inherently impossible in exercise intervention studies, resulting in “some concern” in the “blinding” domain of the RoB assessment. The certainty of evidence for all the endpoints according to the GRADE approach was evaluated, and the results are displayed in Table S3.

All the forest plots, including the heterogeneity test results, as well as all the funnel plots showing the risk of publication bias, can be found in Figures S1–S4.

## 4. Discussion

The potential benefits of a structured exercise program for various aspects of QoL, physical fitness, and overall health in PC patients is a highly relevant area of research. A recent study identified “postoperative rehabilitation addressing physical fitness recovery” as one of six most important criteria that patients who underwent RP expected from a high-quality hospital with a focus on PC [59], highlighting the importance of developing evidence-based exercise interventions.

In this systematic review, we analyzed 30 RCTs, encompassing 38 intervention groups and a total of 2216 participants. To our knowledge, our meta-analysis is the first to separately assess the effects of different exercise modalities, i.e., RT, AT, or a combination of both, on both physical outcomes (body composition, fitness, laboratory markers), psychological outcomes, and QoL. Several key findings can be derived from this analysis.

First, combined RT and AT significantly improved QoL, particularly in the domains of fatigue, cognitive function, sexual function, and urinary symptoms. However, other aspects, like pain, bowel symptoms, hormonal treatment-related symptoms, and physical function did not show significant improvements. Due to a lack of studies specifically implementing RT or AT alone, we were unable to draw definitive conclusions regarding their individual effects on QoL.

Second, body composition—specifically, increases in LBM and decreases in body fat percentage—along with upper and lower body strength and aerobic capacity, can be improved in PC patients through RT, with or without the addition of AT. Our findings suggest that adding AT to an RT program does not offer additional benefits in these areas. However, due to limited data on the effects of AT-only interventions on body composition and fitness parameters, we were unable to quantify its isolated impact, leaving this as an important area for future research.

Third, RT, with or without AT, does not appear to affect blood lipids or PSA levels, which is consistent with previous research.

RT has consistently been shown to improve upper and lower body strength [21,22,23,24,26], as well as LBM, although a few reviews did not detect a significant effect on LBM [22,26]. It is well-known that RT and AT improve fitness and body composition in the general population, and our results confirm that PC and its treatments do not interfere with these positive effects. Regarding QoL, our findings support and expand on the systematic review and meta-analysis by Yang et al. (2017), who demonstrated that AT and/or RT improve cancer-related fatigue and overall QoL in PC patients receiving ADT [27]. Since our meta-analysis included patients at various disease stages and under different therapies, this conclusion can be extended to the broader PC patient population. Additionally, we demonstrated that PC patients particularly benefit from combined RT and AT in two QoL subcategories: sexual function and urinary symptoms.

Since ADT is the most common treatment used among patients in the studies included in this meta-analysis, it was crucial to assess how physical activity influences metabolic changes, like increased cholesterol levels, linked to this treatment. Other reviews have found significant improvements in blood lipids with exercise. For instance, combined AT and RT improved TAG, HDL, and total cholesterol in overweight breast cancer patients or survivors [60]. Another review showed improvements in LDL, HDL, and total cholesterol in patients with hypertension [61], while a review in healthy individuals found improvements in TAG, LDL, and total cholesterol with anaerobic exercise, and RT improved TAG [62]. In our analysis, we did not observe significant changes in lipid levels with combined RT and AT. This may be due to the varying therapies in the study populations; ADT is known to negatively impact blood lipid profiles [63], while EBRT and RP do not. Another factor could be the short intervention durations (<6 months) in the studies we reviewed. While lipid profiles can improve quickly with exercise and diet [64], longer interventions (> 6 months) may be needed for robust changes [65]. Additionally, the studies analyzed may not have adjusted for comorbidities or comedications (e.g., statin therapy), or reported fasting status. Finally, the small sample size (five studies) may have limited the ability to detect significant improvements in lipid profiles.

In this study, we analyzed PSA as an established oncological marker in PC patients. While prior research on PC patients under AS revealed positive effects of AT on PSA levels and PSA doubling time [17,66,67], other trials investigating RT, AT, or combined RT/AT in patients undergoing active therapy found no significant changes in PSA levels [24], which we can confirm in this meta-analysis. Since our analysis included patients across different tumor stages and therapies, identifying clear trends in PSA kinetics under exercise interventions remains difficult. Some research groups have explored new oncological biomarkers for PC. The INTERVAL-GAP4 trial found increased serum levels of the myokines secreted protein acidic and rich in cysteine (SPARC) and oncostatin M (OSM) after 6 months of combined RT and AT, suggesting that these may play a role in exercise-induced cancer suppression [20,68]. Another study, by Gazova et al. (2019), reported elevated plasma levels of miRNA-1 and miRNA-133a after a mixed AT/RT intervention, which are believed to act as tumor suppressors in several cancers, including PC [51]. Future studies should focus on further validating exercise-responsive biomarkers like OSM, SPARC, miRNA-1, and miRNA-133a, which have demonstrated relevance in cancer biology. Another strategy would be to integrate these biomarkers in broader multi-omics studies (e.g., transcriptomics, proteomics, metabolomics) to provide a comprehensive view of the exercise-induced molecular landscape, identifying synergistic pathways and novel targets for intervention. Standardized protocols for measuring and analyzing these biomarkers should be established (e.g., timing of blood sampling to exercise, miRNA profiling techniques) to ensure reproducibility and comparability. To facilitate translation into clinical practice, prospective trials could evaluate these biomarkers as predictive or prognostic markers for exercise efficacy.

Due to the limited number of studies implementing RT or AT alone, this review’s aim to separate the effects of these exercise regimens could not be fully achieved. However, VO_2_peak improved equally well or even slightly better in studies using only RT (effect = 2.3 mL/kg/min) compared to those implementing both RT and AT (effect = 1.3 mL/kg/min). VO_2_peak, a marker of aerobic capacity, depends on maximal cardiac output and the maximal arteriovenous oxygen difference. Although AT traditionally improves stroke volume and oxygen extraction by muscle tissue, RT, especially when performed in circuits with short rest periods, can also enhance VO_2_peak along with the strength improvements, particularly in older populations [69,70,71,72]. This highlights the cardiovascular benefits of RT, while AT has only a limited impact on muscle size and strength [73,74,75,76].

The RoB assessment resulted in a rating of “low risk” or “some concern” for all the included studies. The GRADE assessment supported the robustness of the findings, indicating high-to-moderate certainty of evidence, further strengthening the confidence in the reported outcomes. The RoB domain “blinding” was consistently classified as “some concern”, reflecting the inherent impossibility of blinding in exercise intervention trials. The lack of blinding may introduce bias, particularly in QoL questionnaires, as the participants in exercise groups might perceive outcomes such as pain, urinary frequency, erectile function, or social participation more positively than they actually are, associating their participation in the intervention with the expected benefits. We do not, however, consider this a limitation, since these questionnaires inherently capture subjective perceptions, and any placebo effect associated with the intervention contributes to the overall benefit experienced by the participants.

Several limitations of this systematic review must be acknowledged. Many of the included RCTs had small sample sizes, often being pilot or feasibility studies [20,28,46,51]. Furthermore, the small number of RCTs for several outcomes, such as BMI, body fat percentage, and laboratory parameters, limits the ability to draw robust conclusions. This lack of data restricts the statistical power and may introduce bias, limiting the generalizability of these findings.

The variability of exercise interventions across the studies represents another limitation. Different combinations of RT and AT were used, with significant variations in training duration, intensity, and frequency. Some studies also included additional factors, such as protein or vitamin supplementation, potentially confounding the results. This heterogeneity makes it challenging to isolate the effects of RT or AT alone, or the synergistic impact of combined training. Additionally, the lack of consistency in how the training outcomes were measured further complicates the ability to compare results across studies.

The patient populations in the reviewed studies were diverse, covering different stages of PC and various treatments. This heterogeneity complicates the interpretation of the results, as the effects of exercise may vary depending on cancer stage or concurrent treatment. Importantly, several studies did not specify the disease stage, limiting the ability to perform stratified analyses or draw conclusions about the effectiveness of exercise interventions in specific subgroups. The stratification of the study groups according to the stage of advancement of the participants’ disease or the therapy undertaken mode would improve our understanding of how exercise influences tumors progression, and specifically addresses treatment-related side-effects such as bowel symptoms, in patients after EBRT, or pain, in patients with metastatic bone lesions.

While this review identified significant improvements in QoL and certain fitness parameters, these effects were not consistently observed across all the studies, particularly for isolated RT or AT interventions. The lack of meta-analyses for AT-only interventions due to the limited data reveals a critical gap in the literature and underscores the need for more RCTs focusing on aerobic exercise in PC patients. There are several physiological and practical reasons explaining the scarcity of isolated AT studies. First, in the attempt to counter musculoskeletal side effects of the disease and its treatment, such as decreased LBM and muscle strength, RT is often the preferred modality. Second, since PC patients are often older and carry a variety of comorbidities, further training objectives include fall prevention, bone health, and functional everyday capacity, which are mainly reflected in the methods of strength, mobility, and coordination training. AT plays a subordinate role for this goal, especially as RT already represents an aerobic stimulus when fitness levels are low. Third, the feasibility of AT depends heavily on the equipment available. Without access to ergometers, often only outdoor-based modalities remain, which, apart from being weather-dependent, are difficult to implement on a regular basis, depending on the patient’s mobility. RT, however, can be performed with minimal equipment at a fixed location, including the home, and it is easily adapted to the fitness level of the individual.

The high heterogeneity observed in some outcomes, particularly on fitness tests (e.g., leg press, chest press), also limits the reliability of these findings. This variability may result from differences in how fitness was assessed (e.g., 1-RM vs. 3-RM tests), but it also suggests other unmeasured factors influencing the results. However, regardless of how physical strength was assessed, improvements have consistently been shown. Standardizing fitness tests in future studies could not only reduce measurement bias due to a reduction in the variability in the testing methodologies; they would also enhance comparability across studies, strengthening the statistical power of future meta-analyses, allowing for more robust conclusions and, thus, facilitating translation into clinical practice.

One limitation potentially affecting the statistical validity of our analyses is the need for manual calculation of the IE and confidence intervals due to the inconsistent reporting of the mean pre–post differences and the IE. These retrospective calculations may have introduced bias when sample sizes for each outcome were missing and no confounder adjustments were made.

Although the short-term benefits of exercise are well-established, long-term (>6–12 months) outcomes—both oncological (e.g., biochemical progression, survival) and in general health (e.g., blood markers, body composition)—have been rarely and inconsistently studied. The short duration of most interventions and follow-up periods limits the interpretation of outcomes. The average intervention length was 20.6 weeks, and only a few of the studies provided long-term follow-up data to evaluate the sustainability of the benefits. This raises questions about whether the improvements in QoL, body composition, or fitness can be maintained over time, especially given the progressive nature of PC and its treatment-related side effects.

Adherence to prescribed interventions was not consistently reported across the studies, making it difficult to determine whether the observed effects were due to the interventions or other factors, like varying adherence. This also raises questions about the feasibility of implementing these exercise regimens in real-world clinical settings.

Of the 10 studies lasting a minimum of half a year (26 weeks), 7 reported on adherence [35,41,42,45,47,50,58]. The measurement of adherence was via the attendance rate for prescribed training sessions, and ranged from 49% [47] to 94% [45], with a mean of 69%. Supervised sessions generally had a higher adherence rate than unsupervised sessions. Interestingly, the highest adherence rate, of 94%, was reported by Ashton et al. (2021), who implemented a thrice-weekly RT regime using resistance band exercises, in which supervision was tapered in a structured way. This demonstrates that an autonomous implementation of a structured training program is realistic, provided that adequate instruction is given at the start of the intervention.

Unfortunately, only a few studies conducted follow-up measurements to assess the sustainability of the exercise effects. Ax et al. (2022) reported that the increased physical activity levels, as well as the positive effects on QoL, were maintained one year after the end of the 6-month training intervention [35]. In the study by Galvão et al. (2021), the RT group, which trained for 12 months under constant supervision, achieved sustained QoL and fitness improvements, whereas the mixed RT/AT group, for which the RT part was unsupervised after 6 months, lost some of the QoL improvements, while the fitness parameters stalled [15]. Finally, Newton et al. (2019) reported strength improvements after 6 and 12 months of RT, but hip bone mineral density and appendicular skeletal muscle mass where only improved after 6 months, with no significant difference in relation to the control group after 12 months [42].

The main conclusion that can be derived from those results is that the long-term sustainability of training effects is directly related to adherence to regular structured exercise, and efforts should be undertaken to increase adherence in future exercise studies, e.g., through the approach portrayed by Ashton et al. (2021) [45].

Finally, the eligibility criteria applied for this review excluded studies using other measures of QoL and fitness. Common QoL questionnaires beyond QLQ-C30 and QLQ-PR25 include FACT-P [37,39,45,48,49,50,77,78,79], FACIT-F [38,39,42,77,80], EPIC-26 [28], and SF-36 [11,12,40,46]. Although some of these questionnaires explore some selected domains in more detail (e.g., the emotional aspects in FACT-P, the impact of fatigue on functional activity and social engagement, in FACIT-F, and general health and its impact on everyday life, in EPIC-26 and SF-36), QLQ-C30 and QLQ-PR25 provide a good overview of QoL in PC patients. It is improbable that the results would differ greatly when including other QoL questionnaires in the analysis. Regarding fitness parameters, we only included upper and lower body strength tests, as well as VO_2_peak, although other fitness tests, like the 6-min walk test [36,37,38,51,77], timed up and go test [12,37,43,46,47,55], 30 s chair rising test [44,45,47,56,58], 400 m walk test [52,56,58,81], and stair climbing test [11,40,44,58], are also common. Therefore, we may have missed additional positive effects on QoL and fitness in this review.

## 5. Conclusions

Exercise programs combining RT and AT provide significant benefits for QoL, physical fitness, and body composition in patients with PC. Although the short-term benefits are well-documented, the sustainability of these improvements over time is uncertain.

The impact of AT alone remains unclear, and further research is needed to explore its effects, as well as the long-term benefits of exercise on oncological outcomes like biochemical progression and survival. Future studies should investigate how exercise influences oncological biomarkers beyond PSA and health markers such as blood lipids. Stratifying patients by disease stage and treatment regimen will help to clarify the specific effects of exercise in different PC subgroups.

Addressing these gaps will help to optimize exercise interventions for PC patients, improving both long-term outcomes and overall well-being.

## Figures and Tables

**Table 1:** Characteristics of included studies [11,12,15,16,20,28,35,36,37,38,39,40,41,42,43,44,45,46,47,48,49,50,51,52,53,54,55,56,57,58].

First Author	Year	Sample Size		Mean Age +/− SD (Years)		Disease Stage	Therapy	Intervention Type	DUR	FRQ	Super- Vision	Outcome Measures			
		INT	CON	INT	CON							Body Composition	Fitness	QoL	Laboratory Parameters
Livingston	2015	54	93	66.9 ± 8.2	64.7 ± 8.7	Localized, locally advanced	RP + EBRT + Systemic therapy	RT + AT	12	3	Mixed ^#^			QLQ-C30	
Gaskin	2016	54	66	66.8 ± 8.3	66.0 ± 8.1	Localized, locally advanced	RP + EBRT + Systemic therapy	RT + AT	12	3	Mixed	BMI	Chest press, Leg press		
Harrison	2022	13	13	65.7 ± 8.1	64.4 ± 8.3	Non-metastasized, hormone-sensitive	ADT + ARSI	RT + AT	16	3	Supervised	Body weight, LBM, Fat%	Chest press, Leg press, VO_2_peak		
Hojan	2017	36	36	65.7 ± 6.2	67.9 ± 4.9	Localized, locally advanced	EBRT and/or ADT	RT + AT	8	5	Supervised	Body weight, BMI		QLQ-C30, QLQ-PR25	CHOL, TAG, LDL, HDL, PSA
Kim	2022	13	12	72.6 ± 7.0	76.9 ± 7.1	Metastasized	ADT	RT + AT	26	2	Unsupervised	Body weight, BMI, LBM, Fat%			
Mardani	2021	35	36	69.4 ± 5.8	70.4 ± 5.5	Localized, locally advanced	RP and/or EBRT and/or ADT	RT + AT	12	2	Unsupervised			QLQ-C30, QLQ-PR25	
Ndjavera	2020	24	26	71.4 ± 5.4	72.5 ± 4.2	Locally advanced, metastasized	ADT	RT + AT	12	2	Supervised	Body weight	VO_2_ peak		CHOL, TAG, LDL, HDL, PSA
Ax	2022	49	12	58.0 ± 12.0	58.0 ± 11.0	Localized, locally advanced	EBRT and/or ADT	High-intensity RT + AT	26	4	Mixed			QLQ-C30, QLQ-PR25	
		48		58.0 ± 12.0				Low-/medium RT + AT							
Cormie	2015	32	31	69.6 ± 6.5	67.1 ± 7.5	n/a	EBRT and/or ADT	RT + AT	12	2	Supervised	Body weight, LBM, Fat%	Chest press, Leg press, VO_2_peak	QLQ-PR25	CHOL, TAG, LDL, HDL, PSA
Cormie	2013	29	28	69.5 ± 7.3	70.1 ± 7.3	Localized, locally advanced, metastasized	EBRT and/or ADT	RT + AT	12	2	Supervised			QLQ-PR25	
Evans	2021	20	20	69.5 ± 6.6	70.8 ± 10.2	Metastasized	EBRT and/or ADT	RT + AT	8	4–6	Unsupervised		Chest press, Leg extension	QLQ-C30	
Galvão	2018	28	29	69.7 ± 7.6	70.4 ± 9.3	Metastasized	EBRT and/or ADT and/or CTX	RT + AT	12	3	Supervised	LBM	Leg extension		
Galvão	2010	29	28	69.5 ± 7.3	70.1 ± 7.3	Localized, locally advanced, metastasized	EBRT and/or ADT	RT + AT	12	2	Supervised	Body weight, LBM, Fat%	Chest press, Leg press	QLQ-C30	CHOL, TAG, LDL, HDL, PSA
Galvão	2021	50	36	69.1 ± 9.6	69.7 ± 8.4	n/a	ADT	RT + AT	52	5	Supervised		Chest press, Leg press, Seated row, Leg extension ^§^		
		49		68.7 ± 9.3				RT		5	Mixed				
Galvão	2022	28	29	69.7 ± 7.6	70.4 ± 9.3	Metastasized	EBRT and/or ADT and/or CTX	RT + AT	12	3	Supervised			QLQ-PR25	
Schumacher	2021	72	43	67.9 ± 7.8	67.8 ± 7.5	n/a	EBRT + ADT	RT + AT	26	2–3	Supervised		Chest press, Leg press	QLQ-C30, QLQ-PR25	
Wall	2017	50	47	69.1 ± 9.4	69.1 ± 8.4	Localized	ADT	RT + AT	26	2	Supervised	Body weight, LBM, Fat%			CHOL, TAG, LDL, HDL, PSA
Newton	2020	54	50	69.0 ± 6.3	67.5 ± 7.7	n/a	RP and/or EBRT and/or ADT	RT + AT	26	3	Supervised		Chest press, Leg press		
Newton	2019	50	47	69.1 ± 9.4	69.1 ± 8.4	Localized	EBRT and/or ADT with or without ARSI	RT + AT	26	2	Supervised	LBM	Chest press, Leg press		
		57		68.7 ± 9.3				RT		4	Mixed				
Gazova	2019	15	8	69.2 ± 5.8	70.7 ± 7.5	Localized	ADT	RT	16	3	n/a		Chest press, Leg press		PSA
Houben	2023	30	36	73.0 ± 7.0	71.0 ± 7.0	Non-metastasized, metastasized	ADT	RT + protein	20	2	Supervised	Body weight, BMI, LBM, Fat%	VO_2_peak		
		30		71.0 ± 7.0				RT + placebo							
Nilsen	2015	28	30	66.0 ± 6.6	66.0 ± 5.0	Localized	EBRT + ADT	RT	16	3	Supervised	Body weight, BMI, LBM, Fat%	Chest press, Leg press	QLQ-C30	
Ashton	2021	20	22	64.6 ± 6.2	66.9 ± 6.8	Localized	RP	RT	26	3	Mixed	Body weight, BMI, Fat%	VO_2_peak		CHOL, TAG, LDL, HDL
Cormie	2013	10	10	73.1 ± 7.5	71.2 ± 6.9	Metastasized	ADT	RT	12	2	Supervised	LBM, Fat%	Leg press		
Dalla Via	2021	34	36	71.4 ± 5.9	71.1 ± 6.6	Localized, locally advanced, metastasized	ADT	RT	52	3	Mixed	Body weight, LBM	Chest press, Leg press		PSA
Langlais	2023	7	10	n/a		Metastasized. castration- resistant	ADT + CTX	RT	12	3	Unsupervised			QLQ-C30	
		8						AT							
Kenfield	2021	7	8	73.0 ± n/a	72.0 ± n/a	Metastasized, castration- resistant	ADT + CTX	RT	12	3	Unsupervised		Chest press, Leg press		PSA
		8		70.0 ± n/a				AT							
Alberga	2012	40	41	67.1 ± 6.9	65.4 ± 7.6	Localized, locally advanced, metastasized	EBRT and/or ADT	RT	24	3	Supervised	BMI, LBM, Fat%	Chest press, Leg press VO_2_peak		
		40		66.4 ± 6.8				AT							
Segal	2009	40	41	66.4 ± 7.6	65.3 ± 7.6	Localized, locally advanced, metastasized	EBRT and/or ADT	RT	24	3	Supervised	Body weight, Fat%	VO_2_peak		PSA
		40		66.2 ± 6.8				AT							
Sheill	2023	30	31	69.8 ± 7.0	69.9 ± 7.5	Metastasized	EBRT and/or ADT	AT	26	3–6	Mixed	BMI			

Abbreviations: INT: intervention group; CON: control group; SD: standard deviation; DUR: intervention duration (weeks); FRQ: training frequency (sessions/week); QoL: quality of life; RP: radical prostatectomy; ADT: androgen deprivation therapy; EBRT: external beam radiotherapy; CTX: chemotherapy; ARSI: androgen receptor signaling inhibitor; RT: resistance training; AT: aerobic training; BMI: body mass index; LBM: lean body mass; Fat%: body fat percentage; CHOL: total cholesterol; TAG: triglycerides; LDL: low-density lipoprotein; HDL: high-density lipoprotein; PSA: prostate-specific antigen; n/a: not reported. # Mixed: either only some training sessions in a week were supervised, or supervision was tapered over the time course of the intervention (e.g., supervised in the first weeks, than unsupervised). § In this study, an average strength composite of chest press, leg press, seated row, and leg extension is reported.

## Data Availability

The data presented in this study are available on request from Benazir Enzinger at benazir.enzinger@med.uni-muenchen.de.

## Supplementary Materials

The following supporting information can be downloaded at: https://www.mdpi.com/article/10.3390/cancers16244286/s1. Table S1: PICOS Framework; Table S2: Risk of Bias Assessment; Table S3: Evidence Profiles According to GRADE; Table S4: PRISMA 2020 for Abstracts Checklist; Table S5: PRISMA 2020 Checklist; Figures S1 and S2: Forest Plots; Figures S3 and S4: Funnel Plots.

## References

[1] IARC Data Visualization Tools for Exploring the Global Cancer Burden in 2020. https://gco.iarc.fr/today/home.

[2] Resnick M.J., Koyama T., Fan K.-H., Albertsen P.C., Goodman M., Hamilton A.S., Hoffman R.M., Potosky A.L., Stanford J.L., Stroup A.M. (2013). Long-Term Functional Outcomes after Treatment for Localized Prostate Cancer. N. Engl. J. Med..

[3] Stone P., Hardy J., Huddart R., A’Hern R., Richards M. (2000). Fatigue in Patients with Prostate Cancer Receiving Hormone Therapy. Eur. J. Cancer.

[4] Basaria S., Lieb J., Tang A.M., DeWeese T., Carducci M., Eisenberger M., Dobs A.S. (2002). Long-Term Effects of Androgen Deprivation Therapy in Prostate Cancer Patients. Clin. Endocrinol..

[5] Smith M.R., Finkelstein J.S., McGovern F.J., Zietman A.L., Fallon M.A., Schoenfeld D.A., Kantoff P.W. (2002). Changes in Body Composition during Androgen Deprivation Therapy for Prostate Cancer. J. Clin. Endocrinol. Metab..

[6] Chen Y.Z., Chiang P.K., Lin W.R., Chen M., Chow Y.C., Chiu A.W., Tsai W.K. (2019). The Relationship between Androgen Deprivation Therapy and Depression Symptoms in Patients with Prostate Cancer. Aging Male.

[7] DiBlasio C.J., Hammett J., Malcolm J.B., Judge B.A., Womack J.H., Kincade M.C., Ogles M.L., Mancini J.G., Patterson A.L., Wake R.W. (2008). Prevalence and Predictive Factors for the Development of de Novo Psychiatric Illness in Patients Receiving Androgen Deprivation Therapy for Prostate Cancer. Can. J. Urol..

[8] Bobjer J., Katrinaki M., Tsatsanis C., Lundberg Giwercman Y., Giwercman A.L. (2013). Negative Association between Testosterone Concentration and Inflammatory Markers in Young Men: A Nested Cross-Sectional Study. PLoS ONE.

[9] Stockler M.R., Martin A.J., Davis I.D., Dhillon H.M., Begbie S.D., Chi K.N., Chowdhury S., Coskinas X., Frydenberg M., Hague W.E. (2022). Health-Related Quality of Life in Metastatic, Hormone-Sensitive Prostate Cancer: ENZAMET (ANZUP 1304), an International, Randomized Phase III Trial Led by ANZUP. J. Clin. Oncol..

[10] Kinsey E.N., Zhang T., Armstrong A.J. (2020). Metastatic Hormone-Sensitive Prostate Cancer: A Review of the Current Treatment Landscape. Cancer J..

[11] Galvão D.A., Taaffe D.R., Spry N., Joseph D., Newton R.U. (2010). Combined Resistance and Aerobic Exercise Program Reverses Muscle Loss in Men Undergoing Androgen Suppression Therapy for Prostate Cancer without Bone Metastases: A Randomized Controlled Trial. J. Clin. Oncol..

[12] Galvão D.A., Taaffe D.R., Spry N., Cormie P., Joseph D., Chambers S.K., Chee R., Peddle-Mcintyre C.J., Hart N.H., Baumann F.T. (2018). Exercise Preserves Physical Function in Prostate Cancer Patients with Bone Metastases. Med. Sci. Sports Exerc..

[13] Galvão D.A., Nosaka K., Taaffe D.R., Spry N., Kristjanson L.J., McGuigan M.R., Suzuki K., Yamaya K., Newton R.U. (2006). Resistance Training and Reduction of Treatment Side Effects in Prostate Cancer Patients. Med. Sci. Sports Exerc..

[14] Winters-Stone K.M., Dobek J.C., Bennett J.A., Dieckmann N.F., Maddalozzo G.F., Ryan C.W., Beer T.M. (2015). Resistance Training Reduces Disability in Prostate Cancer Survivors on Androgen Deprivation Therapy: Evidence from a Randomized Controlled Trial. Arch. Phys. Med. Rehabil..

[15] Galvão D.A., Newton R.U., Chambers S.K., Spry N., Joseph D., Gardiner R.A., Fairman C.M., Taaffe D.R. (2021). Psychological Distress in Men with Prostate Cancer Undertaking Androgen Deprivation Therapy: Modifying Effects of Exercise from a Year-Long Randomized Controlled Trial. Prostate Cancer Prostatic Dis..

[16] Cormie P., Newton R.U., Taaffe D.R., Spry N., Joseph D., Akhlil Hamid M., Galvão D.A. (2013). Exercise Maintains Sexual Activity in Men Undergoing Androgen Suppression for Prostate Cancer: A Randomized Controlled Trial. Prostate Cancer Prostatic Dis..

[17] Kang D.-W., Fairey A.S., Boulé N.G., Field C.J., Wharton S.A., Courneya K.S. (2021). Effects of Exercise on Cardiorespiratory Fitness and Biochemical Progression in Men with Localized Prostate Cancer Under Active Surveillance. JAMA Oncol..

[18] Shao W., Zhang H., Qi H., Zhang Y. (2022). The Effects of Exercise on Body Composition of Prostate Cancer Patients Receiving Androgen Deprivation Therapy: An Update Systematic Review and Meta-Analysis. PLoS ONE.

[19] Nilsen T.S., Johansen S.H., Thorsen L., Fairman C.M., Wisløff T., Raastad T. (2022). Does Androgen Deprivation for Prostate Cancer Affect Normal Adaptation to Resistance Exercise?. Int. J. Environ. Res. Public Health.

[20] Kim J.S., Taaffe D.R., Galvão D.A., Hart N.H., Gray E., Ryan C.J., Kenfield S.A., Saad F., Newton R.U. (2022). Exercise in Advanced Prostate Cancer Elevates Myokine Levels and Suppresses In-Vitro Cell Growth. Prostate Cancer Prostatic Dis..

[21] Bourke L., Smith D., Steed L., Hooper R., Carter A., Catto J., Albertsen P.C., Tombal B., Payne H.A., Rosario D.J. (2016). Exercise for Men with Prostate Cancer: A Systematic Review and Meta-Analysis. Eur. Urol..

[22] Chen Z., Zhang Y., Lu C., Zeng H., Schumann M., Cheng S. (2019). Supervised Physical Training Enhances Muscle Strength but Not Muscle Mass in Prostate Cancer Patients Undergoing Androgen Deprivation Therapy: A Systematic Review and Meta-Analysis. Front. Physiol..

[23] Keilani M., Hasenoehrl T., Baumann L., Ristl R., Schwarz M., Marhold M., Sedghi Komandj T., Crevenna R. (2017). Effects of Resistance Exercise in Prostate Cancer Patients: A Meta-Analysis. Support. Care Cancer.

[24] Lopez P., Taaffe D.R., Newton R.U., Galvão D.A. (2021). Resistance Exercise Dosage in Men with Prostate Cancer: Systematic Review, Meta-Analysis, and Meta-Regression. Med. Sci. Sports Exerc..

[25] Ying M., Zhao R., Jiang D., Gu S., Li M. (2018). Lifestyle Interventions to Alleviate Side Effects on Prostate Cancer Patients Receiving Androgen Deprivation Therapy: A Meta-Analysis. Jpn. J. Clin. Oncol..

[26] Yunfeng G., Weiyang H., Xueyang H., Yilong H., Xin G. (2017). Exercise Overcome Adverse Effects among Prostate Cancer Patients Receiving Androgen Deprivation Therapy: An Update Meta-Analysis. Medicine.

[27] Yang B., Wang J., Yang B., Wang J. (2017). Effects of Exercise on Cancer-Related Fatigue and Quality of Life in Prostate Cancer Patients Undergoing Androgen Deprivation Therapy: A Meta-Analysis of Randomized Clinical Trials. Chin. Med. Sci. J..

[28] Langlais C.S., Chen Y.H., Van Blarigan E.L., Chan J.M., Ryan C.J., Zhang L., Borno H.T., Newton R.U., Luke A., Bang A.S. (2023). Quality of Life for Men with Metastatic Castrate-Resistant Prostate Cancer Participating in an Aerobic and Resistance Exercise Pilot Intervention. Urol. Oncol..

[29] Page M.J., McKenzie J.E., Bossuyt P.M., Boutron I., Hoffmann T.C., Mulrow C.D., Shamseer L., Tetzlaff J.M., Akl E.A., Brennan S.E. (2021). The PRISMA 2020 Statement: An Updated Guideline for Reporting Systematic Reviews. BMJ.

[30] Aaronson N.K., Ahmedzai S., Bergman B., Bullinger M., Cull A., Duez N.J., Filiberti A., Flechtner H., Fleishman S.B., Haes J.C.J.M.D. (1993). The European Organization for Research and Treatment of Cancer QLQ-C30: A Quality-of-Life Instrument for Use in International Clinical Trials in Oncology. J. Natl. Cancer Inst..

[31] Chu D., Popovic M., Chow E., Cella D., Beaumont J.L., Lam H., Nguyen J., Di Giovanni J., Pulenzas N., Bedard G. (2014). Development, Characteristics and Validity of the EORTC QLQ-PR25 and the FACT-P for Assessment of Quality of Life in Prostate Cancer Patients. J. Comp. Eff. Res..

[32] Guyatt G., Oxman A.D., Akl E.A., Kunz R., Vist G., Brozek J., Norris S., Falck-Ytter Y., Glasziou P., Debeer H. (2011). GRADE Guidelines: 1. Introduction—GRADE Evidence Profiles and Summary of Findings Tables. J. Clin. Epidemiol..

[33] Montgomery P., Movsisyan A., Grant S.P., Macdonald G., Rehfuess E.A. (2019). Considerations of Complexity in Rating Certainty of Evidence in Systematic Reviews: A Primer on Using the GRADE Approach in Global Health. BMJ Glob. Health.

[34] Hackshaw-Mcgeagh L.E., Penfold C., Shingler E., Robles L.A., Perks C.M., Holly J.M.P., Rowe E., Koupparis A., Bahl A., Persad R. (2019). Phase II Randomised Control Feasibility Trial of a Nutrition and Physical Activity Intervention after Radical Prostatectomy for Prostate Cancer. BMJ Open.

[35] Ax A.K., Johansson B., Lyth J., Nordin K., Börjeson S. (2022). Short- and Long-Term Effect of High versus Low-to-Moderate Intensity Exercise to Optimise Health-Related Quality of Life after Oncological Treatment—Results from the Phys-Can Project. Support. Care Cancer.

[36] Gaskin C.J., Fraser S.F., Owen P.J., Craike M., Orellana L., Livingston P.M. (2016). Fitness Outcomes from a Randomised Controlled Trial of Exercise Training for Men with Prostate Cancer: The ENGAGE Study. J. Cancer Surviv..

[37] Harrison M.R., Davis P.G., Khouri M.G., Bartlett D.B., Gupta R.T., Armstrong A.J., McNamara M.A., Zhang T., Anand M., Onyenwoke K. (2022). A Randomized Controlled Trial Comparing Changes in Fitness with or without Supervised Exercise in Patients Initiated on Enzalutamide and Androgen Deprivation Therapy for Non-Metastatic Castration-Sensitive Prostate Cancer (EXTEND). Prostate Cancer Prostatic Dis..

[38] Hojan K., Kwiatkowska-Borowczyk E., Leporowska E., Milecki P. (2017). Inflammation, Cardiometabolic Markers, and Functional Changes in Men with Prostate Cancer. A Randomized Controlled Trial of a 12-month Exercise Program. Pol. Arch. Intern. Med..

[39] Ndjavera W., Orange S.T., O’Doherty A.F., Leicht A.S., Rochester M., Mills R., Saxton J.M. (2020). Exercise-Induced Attenuation of Treatment Side-Effects in Patients with Newly Diagnosed Prostate Cancer Beginning Androgen-Deprivation Therapy: A Randomised Controlled Trial. BJU Int..

[40] Cormie P., Galvão D.A., Spry N., Joseph D., Chee R., Taaffe D.R., Chambers S.K., Newton R.U. (2015). Can Supervised Exercise Prevent Treatment Toxicity in Patients with Prostate Cancer Initiating Androgen-Deprivation Therapy: A Randomised Controlled Trial. BJU Int..

[41] Wall B.A., Galvão D.A., Fatehee N., Taaffe D.R., Spry N., Joseph D., Hebert J.J., Newton R.U. (2017). Exercise Improves VO2max and Body Composition in Androgen Deprivation Therapy-Treated Prostate Cancer Patients. Med. Sci. Sports Exerc..

[42] Newton R.U., Galvão D.A., Spry N., Joseph D., Chambers S.K., Gardiner R.A., Wall B.A., Bolam K.A., Taaffe D.R. (2019). Exercise Mode Specificity for Preserving Spine and Hip Bone Mineral Density in Prostate Cancer Patients. Med. Sci. Sports Exerc..

[43] Houben L.H.P., Overkamp M., Van Kraaij P., Trommelen J., Van Roermund J.G.H., De Vries P., De Laet K., Van Der Meer S., Mikkelsen U.R., Verdijk L.E.X.B. (2023). Resistance Exercise Training Increases Muscle Mass and Strength in Prostate Cancer Patients on Androgen Deprivation Therapy. Med. Sci. Sports Exerc..

[44] Nilsen T.S., Raastad T., Skovlund E., Courneya K.S., Langberg C.W., Lilleby W., Fosså S.D., Thorsen L. (2015). Effects of Strength Training on Body Composition, Physical Functioning, and Quality of Life in Prostate Cancer Patients during Androgen Deprivation Therapy. Acta Oncol..

[45] Ashton R.E., Aning J.J., Tew G.A., Robson W.A., Saxton J.M. (2021). Supported Progressive Resistance Exercise Training to Counter the Adverse Side Effects of Robot-Assisted Radical Prostatectomy: A Randomised Controlled Trial. Support. Care Cancer.

[46] Cormie P., Newton R.U., Spry N., Joseph D., Taaffe D.R., Galvão D.A. (2013). Safety and Efficacy of Resistance Exercise in Prostate Cancer Patients with Bone Metastases. Prostate Cancer Prostatic Dis..

[47] Dalla Via J., Owen P.J., Daly R.M., Mundell N.L., Livingston P.M., Rantalainen T., Foulkes S.J., Millar J.L., Murphy D.G., Fraser S.F. (2021). Musculoskeletal Responses to Exercise Plus Nutrition in Men with Prostate Cancer on Androgen Deprivation: A 12-Month RCT. Med. Sci. Sports Exerc..

[48] Alberga A.S., Segal R.J., Reid R.D., Scott C.G., Sigal R.J., Khandwala F., Jaffey J., Wells G.A., Kenny G.P. (2012). Age and Androgen-Deprivation Therapy on Exercise Outcomes in Men with Prostate Cancer. Support. Care Cancer.

[49] Segal R.J., Reid R.D., Courneya K.S., Sigal R.J., Kenny G.P., Prud’Homme D.G., Malone S.C., Wells G.A., Scott C.G., Slovinec D’Angelo M.E. (2009). Randomized Controlled Trial of Resistance or Aerobic Exercise in Men Receiving Radiation Therapy for Prostate Cancer. J. Clin. Oncol..

[50] Sheill G., Brady L., Hayes B., Baird A.M., Guinan E., Vishwakarma R., Brophy C., Vlajnic T., Casey O., Murphy V. (2023). ExPeCT: A Randomised Trial Examining the Impact of Exercise on Quality of Life in Men with Metastatic Prostate Cancer. Support. Care Cancer.

[51] Gazova A., Samakova A., Laczo E., Hamar D., Polakovicová M., Jurikova M., Kyselovic J. (2019). Clinical Utility of MiRNA-1, MiRNA-29g and MiRNA-133s Plasma Levels in Prostate Cancer Patients with High-Intensity Training after Androgen-Deprivation Therapy. Physiol. Res..

[52] Kenfield S.A., Van Blarigan E.L., Panchal N., Bang A., Zhang L., Graff R.E., Chen Y.H., Ryan C.J., Luke A., Newton R.U. (2021). Feasibility, Safety, and Acceptability of a Remotely Monitored Exercise Pilot CHAMP: A Clinical Trial of High-Intensity Aerobic and Resistance Exercise for Metastatic Castrate-Resistant Prostate Cancer. Cancer Med..

[53] Livingston P.M., Craike M.J., Salmon J., Courneya K.S., Gaskin C.J., Fraser S.F., Mohebbi M., Broadbent S., Botti M., Kent B. (2015). Effects of a Clinician Referral and Exercise Program for Men Who Have Completed Active Treatment for Prostate Cancer: A Multicenter Cluster Randomized Controlled Trial (ENGAGE). Cancer.

[54] Mardani A., Pedram Razi S., Mazaheri R., Haghani S., Vaismoradi M. (2021). Effect of the Exercise Programme on the Quality of Life of Prostate Cancer Survivors: A Randomized Controlled Trial. Int. J. Nurs. Pract..

[55] Evans H.E.L., Galvão D.A., Forbes C.C., Girard D., Vandelanotte C., Newton R.U., Vincent A.D., Wittert G., Kichenadasse G., Chambers S. (2021). Acceptability and Preliminary Efficacy of a Web- and Telephone-Based Personalised Exercise Intervention for Individuals with Metastatic Prostate Cancer: The ExerciseGuide Pilot Randomised Controlled Trial. Cancers.

[56] Schumacher O., Galvão D.A., Taaffe D.R., Spry N., Joseph D., Tang C., Chee R., Newton R.U. (2021). Effect of Exercise Adjunct to Radiation and Androgen Deprivation Therapy on Patient-Reported Treatment Toxicity in Men with Prostate Cancer: A Secondary Analysis of 2 Randomized Controlled Trials. Pract. Radiat. Oncol..

[57] Galvão D.A., Taaffe D.R., Chambers S.K., Fairman C.M., Spry N., Joseph D., Newton R.U. (2022). Exercise Intervention and Sexual Function in Advanced Prostate Cancer: A Randomised Controlled Trial. BMJ Support. Palliat. Care.

[58] Newton R.U., Galvão D.A., Spry N., Joseph D., Chambers S.K., Gardiner R.A., Hayne D., Taaffe D.R. (2020). Timing of Exercise for Muscle Strength and Physical Function in Men Initiating ADT for Prostate Cancer. Prostate Cancer Prostatic Dis..

[59] Wolff I., Burchardt M., Peter J., Thomas C., Sikic D., Fiebig C., Promnitz S., Hoschke B., Burger M., Schnabel M.J. (2023). Patient’s Desire and Real Availability Concerning Supportive Measures Accompanying Radical Prostatectomy: Differences between Certified Prostate Cancer Centers and Non-Certified Centers Based on Patient-Reported Outcomes within the Cross-Sectional Study Improve. Cancers.

[60] Al-Mhanna S.B., Batrakoulis A., Noor N.M., Mohamed M., Drenowatz C., Irekeola A.A., Afolabi H.A., Gülü M., Alkhamees N.H., Ghazali W.S.W. (2024). Combined Aerobic and Resistance Training Improves Body Composition, Alters Cardiometabolic Risk, and Ameliorates Cancer-Related Indicators in Breast Cancer Patients and Survivors with Overweight/Obesity: A Systematic Review and Meta-Analysis of Randomized Controlled Trials. J. Sports Sci. Med..

[61] Fei X., Li H., Liu M., Li C. (2022). Effect of Exercise on Blood Lipid for Patients with Hypertension: A Network Meta-Analysis. Med. Sci. Sports Exerc..

[62] Buzdagli Y., Tekin A., Eyipinar C.D., Öget F., Siktar E. (2022). The Effect of Different Types of Exercise on Blood Lipid Profiles: A Meta-Analysis of Randomized Controlled Studies. Sci. Sports.

[63] Choi S.M., Kam S.C. (2015). Metabolic Effects of Androgen Deprivation Therapy. Korean J. Urol..

[64] Khalafi M., Sakhaei M.H., Kazeminasab F., Rosenkranz S.K., Symonds M.E. (2023). Exercise Training, Dietary Intervention, or Combined Interventions and Their Effects on Lipid Profiles in Adults with Overweight and Obesity: A Systematic Review and Meta-Analysis of Randomized Clinical Trials. Nutr. Metab. Cardiovasc. Dis..

[65] Candás-Estébanez B., Fernández-Cidón B., Corbella E., Tebé C., Fanlo-Maresma M., Esteve-Luque V., Salas-Salvadó J., Fitó M., Riera-Mestre A., Ros E. (2024). The Impact of the Mediterranean Diet and Lifestyle Intervention on Lipoprotein Subclass Profiles among Metabolic Syndrome Patients: Findings of a Randomized Controlled Trial. Int. J. Mol. Sci..

[66] Hvid T., Lindegaard B., Winding K., Iversen P., Brasso K., Solomon T.P.J., Pedersen B.K., Hojman P. (2016). Effect of a 2-Year Home-Based Endurance Training Intervention on Physiological Function and PSA Doubling Time in Prostate Cancer Patients. Cancer Causes Control..

[67] Lee D.J., Byeon J.Y., Park D.H., Oh C.G., Lee J., Choi Y.D., Kang D.W., An K.Y., Courneya K.S., Lee D.H. (2024). Effects of Exercise during Active Surveillance for Prostate Cancer: A Systematic Review and Meta-Analysis. Support. Care Cancer.

[68] Kim J.S., Galvão D.A., Newton R.U., Gray E., Taaffe D.R. (2021). Exercise-Induced Myokines and Their Effect on Prostate Cancer. Nat. Rev. Urol..

[69] Konzen V., Oliveira M., Schneider R., Wibelinger L. (2024). Impact of Resistant Exercise on the Cardiorespiratory Fitness of the Elderly: A Systematic Review. Concilium.

[70] Smart T.F.F., Doleman B., Hatt J., Paul M., Toft S., Lund J.N., Phillips B.E. (2022). The Role of Resistance Exercise Training for Improving Cardiorespiratory Fitness in Healthy Older Adults: A Systematic Review and Meta-Analysis. Age Ageing.

[71] Lambert C.P., Evans W.J. (2005). Adaptations to Aerobic and Resistance Exercise in the Elderly. Rev. Endocr. Metab. Disord..

[72] Jones L.M., Stoner L., Baldi J.C., McLaren B. (2020). Circuit Resistance Training and Cardiovascular Health in Breast Cancer Survivors. Eur. J. Cancer Care.

[73] Brightwell C.R., Markofski M.M., Moro T., Fry C.S., Porter C., Volpi E., Rasmussen B.B. (2019). Moderate-Intensity Aerobic Exercise Improves Skeletal Muscle Quality in Older Adults. Transl. Sports Med..

[74] Winters-Stone K.M., Torgrimson-Ojerio B., Dieckmann N.F., Stoyles S., Mitri Z., Luoh S.W. (2022). A Randomized-Controlled Trial Comparing Supervised Aerobic Training to Resistance Training Followed by Unsupervised Exercise on Physical Functioning in Older Breast Cancer Survivors. J. Geriatr. Oncol..

[75] Aires I., Duarte J.A., Vitorino R., Moreira-Gonçalves D., Oliveira P., Ferreira R. (2024). Restoring Skeletal Muscle Health through Exercise in Breast Cancer Patients and after Receiving Chemotherapy. Int. J. Mol. Sci..

[76] Andersen M.F., Midtgaard J., Bjerre E.D. (2022). Do Patients with Prostate Cancer Benefit from Exercise Interventions? A Systematic Review and Meta-Analysis. Int. J. Environ. Res. Public Health.

[77] Piraux E., Caty G., Renard L., Vancraeynest D., Tombal B., Geets X., Reychler G. (2020). Effects of High-Intensity Interval Training Compared with Resistance Training in Prostate Cancer Patients Undergoing Radiotherapy: A Randomized Controlled Trial. Prostate Cancer Prostatic Dis..

[78] Segal R.J., Reid R.D., Courneya K.S., Malone S.C., Parliament M.B., Scott C.G., Venner P.M., Quinney H.A., Jones L.W., D’Angelo M.E.S. (2016). Resistance Exercise in Men Receiving Androgen Deprivation Therapy for Prostate Cancer. J. Clin. Oncol..

[79] Monga U., Garber S.L., Thornby J., Vallbona C., Kerrigan A.J., Monga T.N., Zimmermann K.P. (2007). Exercise Prevents Fatigue and Improves Quality of Life in Prostate Cancer Patients Undergoing Radiotherapy. Arch. Phys. Med. Rehabil..

[80] Newton R.U., Mavropalias G., Fragala M.S., Kraemer W.J., Häkkinen K., Taaffe D.R., Spry N., Joseph D., Galvão D.A. (2021). Radiotherapy before or during Androgen-Deprivation Therapy Does Not Blunt the Exercise-Induced Body Composition Protective Effects in Prostate Cancer Patients: A Secondary Analysis of Two Randomized Controlled Trials. Exp. Gerontol..

[81] Taaffe D.R., Newton R.U., Spry N., Joseph D., Chambers S.K., Gardiner R.A., Wall B.A., Cormie P., Bolam K.A., Galvão D.A. (2017). Effects of Different Exercise Modalities on Fatigue in Prostate Cancer Patients Undergoing Androgen Deprivation Therapy: A Year-Long Randomised Controlled Trial. Eur. Urol..

