# Supplementary material for: The Effect of Resistance and/or Aerobic Training on Quality of Life, Fitness, and Body Composition in Prostate Cancer Patients—A Systematic Review and Meta-Analysis
Source: Cancers (Basel). 2024 Dec 23;16(24):4286. doi: 10.3390/cancers16244286 (PMC11674139; doi:10.3390/cancers16244286)
Supplement: Supplementary file 1 [file cancers-16-04286-s001.zip › cancers-3342101-supplementary.pdf]

Cancers

Supplementary Data.

Supplement to

The Effect of Resistance and/or Aerobic Training on Quality of Life, Fitness, and Body Composition in Prostate Cancer Patients – A Systematic Review and Meta-Analysis

Shimon Kempin, Alexander Buchner, Sarah Frederike Brose, Nina Schmidt-Hegemann, Matthias May, Ingmar Wolff, Anton Kravchuk, Christian Stief, Sabine D. Brookman-May, and Benazir Enzinger

Table of Content

1. Authors 3

2. PROSPERO Registration 4

3. PICOS Framework (Table S1) 5

4. Search Strategy 6

5. Risk of Bias Assessment (Table S2) 7

6. Evidence Profiles According to GRADE (Table S3) 8

7. Forest Plots—Effects of Combined Resistance and Aerobic Training (Figures S1 a-h) 10

8. Forest Plots—Effects of Resistance Training Only (Figures S2 a-h) 13

9. Funnel Plots—Effects of Combined Resistance and Aerobic Training (Figures S3 a-w) 16

10. Funnel Plots—Effects of Resistance Training Only (Figures S4 a-h) 24

11. PRISMA 2020 for Abstracts Checklist (Table S4) 27

12. PRISMA 2020 Checklist (Table S5) 28

References 31

1. Authors

Author	Degree	Affiliation	E-Mail
**Shimon Kempin**	B.Sc.	Department of Urology, LMU University Hospital, Ludwig Maximilian University of Munich, Munich, Germany	s.kempin@campus.lmu.de
**Alexander Buchner**	MD, Prof.	Department of Urology, LMU University Hospital, Ludwig Maximilian University of Munich, Munich, Germany	alexander.buchner@med.uni-muenchen.de
**Sarah Frederike Brose**	MD	Department of Radiation Oncology, LMU University Hospital, Ludwig Maximilian University of Munich, Munich, Germany	sarah.brose@med.uni-muenchen.de
**Nina Schmidt-Hegemann**	MD, Assoc.-Prof.	Department of Radiation Oncology, LMU University Hospital, Ludwig Maximilian University of Munich, Munich, Germany	nina-sophie.hegemann@med.uni-muenchen.de
**Matthias May**	MD, Prof.	Department of Urology, St. Elisabeth Hospital Straubing, Brothers of Mercy Hospital, Straubing, Germany	matthias.may@klinikum-straubing.de
**Ingmar Wolff**	MD	Department of Urology, University Medicine Greifswald, Greifswald, Germany	ingmar.wolff@med.uni-greifswald.de
**Anton Kravchuk**	MD	Department of Urology, St. Elisabeth Hospital Straubing, Brothers of Mercy Hospital, Straubing, Germany	anton.kravchuk@klinikum-straubing.de
**Christian Stief**	MD, Prof.	Department of Urology, LMU University Hospital, Ludwig Maximilian University of Munich, Munich, Germany	christian.stief@med.uni-muenchen.de
**Sabine D. Brookman-May**	MD, Prof.	Department of Urology, LMU University Hospital, Ludwig Maximilian University of Munich, Munich, Germany<br>Aura Biosciences, Boston, MA, USA	sabine.brookman-may@outlook.com
**Benazir Enzinger**	B.Sc., MPP, MD	Department of Urology, LMU University Hospital, Ludwig Maximilian University of Munich, Munich, Germany	benazir.enzinger@med.uni-muenchen.de

2. PROSPERO Registration

This study was registered on the Prospero database (CRD42024552379) on 09/06/2024.

3. PICOS Framework (Table S1)

The research question for our review can be expressed using PICOS (Population, Intervention, Comparator, Outcome, Study design), as follows (Supplementary Table 1):

Table S1. PICOS Framework

Element	Focus
**Clinical Question**	Does structured RT and/or AT training benefit PC patients regarding physiological and psychological parameters?
**Population**	Adult men with different stages of PC undergoing active therapy
**Intervention**	Structured RT and/or AT
**Comparator**	Standard of Care (SOC)
**Outcomes**	QoL (via patient reported outcomes), Body composition, Laboratory parameters (including PSA and general health markers), Fitness parameters (upper and lower body strength, VO_2peak)
**Study Design**	RCTs comparing structured RT and/or AT programs to SOC
**Databases Searched**	PubMed, EMBASE, Cochrane Library
**Timeframe**	Minimum intervention length of 4 weeks, no upper limit
**Geography**	No geographic limit
**Language**	English
**Eligibility Criteria**	- RCTs including patients aged > 18 years at any PC stage (localized, locally advanced, or advanced) who are receiving/have recently received active therapy (RP, EBRT, or systemic treatment).<br>- Patients have to be provided with a structured RT and/or AT program that can be performed at home or at a centralized facility with or without supervision.
**Exclusion Criteria**	- Case reports, editorials, letters to editors, animal and pediatric studies, study protocols.<br>- Studies without a direct comparison between the intervention and the comparator.<br>- The training program includes only pelvic floor exercises.<br>- Study does not report on any of the mentioned outcomes.<br>- Inclusion of a mixed cancer cohort without reporting separately on the outcomes specific for PC patients.<br>- Participants did not receive any active therapy 12 months prior to the intervention.<br>- Pharmacologically active agents (e.g. metformin) were used in addition to the intervention.<br>- The results were published in a language other than English.

Abbreviations: RT: resistance training, AT: aerobic training, PC: prostate cancer, QoL: quality of life, PSA: prostate-specific antigen, RCTs: randomized controlled trials, RP: radical prostatectomy, EBRT: external beam radiotherapy

4. Search Strategy

The following terms were used for the search of studies meeting the predefined inclusion criteria: "(randomized controlled trial) AND (prostate cancer OR prostatic neoplasms) AND (physical exercise) NOT (pelvic floor exercise)". Databases were systematically explored up to May 2024.

5. Risk of Bias Assessment (Table S2)

Table S2. Risk of bias assessment.

No.	First Author, Year	D1[a]	D2[b]	D3[c]	D4[d]	Overall
**1**	Livingston 2015 [1]	Low risk	Some concern	Low risk	Low risk	Low risk
**2**	Gaskin 2016 [2]	Low risk	Some concern	Low risk	Low risk	Low risk
**3**	Harrison 2022 [3]	Low risk	Some concern	Low risk	Low risk	Low risk
**4**	Hojan 2017 [4]	Low risk	Some concern	Low risk	Low risk	Low risk
**5**	Kim 2022 [5]	Low risk	Some concern	Low risk	Low risk	Low risk
**6**	Mardani 2021 [6]	Low risk	Some concern	Some concern	Low risk	Some concern
**7**	Ndjavera 2020 [7]	Low risk	Some concern	Some concern	Low risk	Some concern
**8**	Ax 2022 [8]	Low risk	Some concern	Low risk	Low risk	Low risk
**9**	Cormie 2015 [9]	Some concern	Some concern	Low risk	Low risk	Some concern
**10**	Cormie 2013a [10]	Low risk	Some concern	Low risk	Low risk	Low risk
**11**	Evans 2021 [11]	Some concern	Some concern	Low risk	Low risk	Some concern
**12**	Galvão 2018 [12]	Low risk	Some concern	Some concern	Low risk	Some concern
**13**	Galvão 2010 [13]	Low risk	Some concern	Low risk	Low risk	Low risk
**14**	Galvão 2021 [14]	Low risk	Some concern	Low risk	Low risk	Low risk
**15**	Galvão 2022 [15]	Low risk	Some concern	Some concern	Low risk	Some concern
**16**	Schumacher 2021 [16]	Some concern	Some concern	Low risk	Low risk	Some concern
**17**	Wall 2017 [17]	Low risk	Some concern	Low risk	Low risk	Low risk
**18**	Newton 2020 [18]	Low risk	Some concern	Some concern	Low risk	Some concern
**19**	Newton 2019 [19]	Low risk	Some concern	Some concern	Low risk	Some concern
**20**	Gazova 2019 [20]	Some concern	Some concern	Low risk	Some concern	Some concern
**21**	Houben 2023 [21]	Some concern	Some concern	Some concern	Low risk	Some concern
**22**	Nilsen 2015 [22]	Low risk	Some concern	Low risk	Low risk	Low risk
**23**	Ashton 2021 [23]	Low risk	Some concern	Some concern	Low risk	Some concern
**24**	Cormie 2013b [24]	Low risk	Some concern	Low risk	Low risk	Low risk
**25**	Dalla Via 2021 [25]	Low risk	Some concern	Low risk	Low risk	Low risk
**26**	Langlais 2023 [26]	Low risk	Some concern	Some concern	Low risk	Some concern
**27**	Kenfield 2021 [27]	Low risk	Some concern	Low risk	Low risk	Low risk
**28**	Alberga 2012 [28]	Some concern	Some concern	Low risk	Low risk	Some concern
**29**	Segal 2009 [29]	Low risk	Some concern	Low risk	Low risk	Low risk
**30**	Sheill 2023 [30]	Low risk	Some concern	Low risk	Low risk	Low risk

[a] Domain 1: randomization process

[b] Domain 2: blinding

[c] Domain 3: incomplete outcome data

[d] Domain 4: selective reporting

6. Evidence Profiles According to GRADE (Table S3)

Table S3 a. Evidence profile of the most clinically relevant endpoints analyzing the effects of combined resistance and aerobic training.

Endpoints	No. of Compa-risons	Sample Size	Certainty Assessment					IE (95% CI)	Certainty
			Risk of Bias[a]	Inconsistency[b]	Indirectness[c]	Imprecision[d]	Publication Bias[e]		
QLQ-C30 Global Health Status	8	611	Low risk	Not serious	Not serious	Not serious	Unlikely	2.4 (0.0 – 4.8)	⊕⊕⊕⊕
QLQ-C30 Fatigue	7	464	Low risk	Not serious	Not serious	Not serious	Unlikely	-8.1 (-12.8 – -3.5)	⊕⊕⊕⊕
QLQ-PR25 Sexual Functioning	5	359	Some concern	Not serious	Not serious	Not serious	Unlikely	10.9 (2.7 – 19.1)	⊕⊕⊕∅
QLQ-PR25 Urinary Symptoms	8	544	Some concern	Not serious	Not serious	Not serious	Unlikely	-3.3 (-6.6 – -0.1)	⊕⊕⊕∅
Lean Body Mass (kg)	7	422	Low risk	Not serious	Not serious	Not serious	Unlikely	0.6 (0.2 – 0.9)	⊕⊕⊕⊕
1-RM Leg Press (kg)	10	765	Some concern	Not serious	Not serious	Not serious	Unlikely	20.2 (13.7 – 26.8)	⊕⊕⊕∅
VO_2peak (ml/kg/min)	3	139	Some concern	Not serious	Not serious	Not serious	Unlikely	1.3 (0.6 – 2.0)	⊕⊕⊕∅
LDL (mmol/l)	5	339	Low risk	Not serious	Not serious	Not serious	Unlikely	0.0 (-0.17 – 0.16)	⊕⊕⊕⊕
PSA (ng/ml)	4	267	Some concern	Not serious	Not serious	Not serious	Unlikely	0.06 (-0.23 – 0.35)	⊕⊕⊕∅

Table S3 b. Evidence profile of the endpoints analyzing the effects of resistance training only.

Endpoints	No. of Compa-risons	Sample Size	Certainty Assessment					IE (95% CI)	Certainty
			Risk of Bias	Inconsistency	Indirectness	Imprecision	Publication Bias[e]		
QLQ-C30 Global Health Status	2	75	Not enough data for analysis						
QLQ-C30 Fatigue	2	75	Not enough data for analysis						
QLQ-PR25 Sexual Functioning	0	0	Not enough data for analysis						
QLQ-PR25 Urinary Symptoms	0	0	Not enough data for analysis						
Lean Body Mass (kg)	7	399	Some concern	Not serious	Not serious	Not serious	Unlikely	0.6 (0.2 – 1.0)	⊕⊕⊕∅
1-RM Leg Press (kg)	8	456	Low risk	Very serious	Not serious	Not serious	Unlikely	18.6 (8.7 – 28.6)	⊕⊕∅∅
VO_2peak (ml/kg/min)	5	270	Some concern	Serious	Not serious	Not serious	Unlikely	2.3 (0.8 – 3.8)	⊕⊕∅∅
LDL (mmol/l)	1	42	Not enough data for analysis						
PSA (ng/ml)	4	189	Low risk	Serious	Not serious	Not serious	Unlikely	-0.1 (-0,9 – 0.8)	⊕⊕⊕∅

Certainty grades:

⊕⊕⊕⊕ High

⊕⊕⊕∅ Moderate

⊕⊕∅∅ Low

⊕∅∅∅ Very Low

No meta-analysis was performed for the effect of aerobic training due to a lack of data. Thus, no certainty of evidence table for this study subgroup was created.

[a] Risk of bias was assessed through systematic analysis of all included studies in regard to the domains “randomization process”, “blinding”, ‘incomplete outcome data’, and ‘selective reporting’.

[b] Inconsistency was assessed using the I^2 statistic. I^2 of < 30% was considered “not serious”, I^2 between 30% and 60% as “serious”, and I^2 of > 60% as “very serious”

[c] Indirectness is a measure of the proximity of the study populations, interventions, comparators, and outcomes to those of the research question. Since we only included studies matching our PICOS criteria (see Supplementary Table 1), indirectness was considered not serious for all outcomes.

[d] Imprecision was assessed by analyzing the width of the pooled effect confidence interval for each outcome.

[e] Publication bias was assessed by visual inspection of the funnel plots, as well as analyzing the Begg and Egger’s test for each outcome (see Figures S3 and S4).

7. Forest Plots—Effects of Combined Resistance and Aerobic Training (Figures S1 a–h)

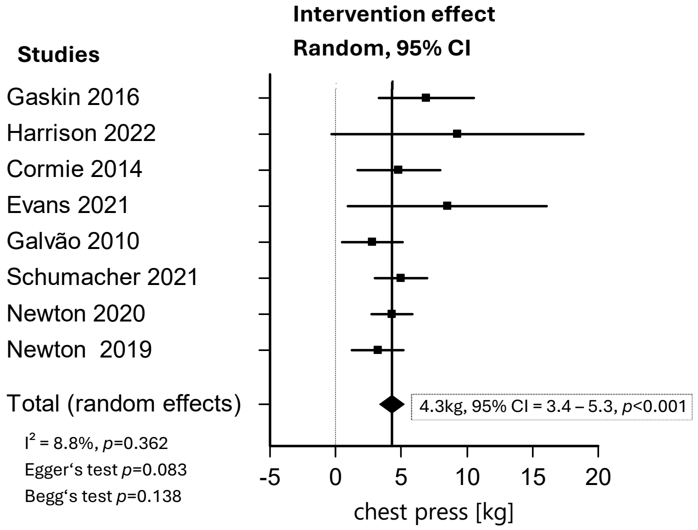

Figure S1 a

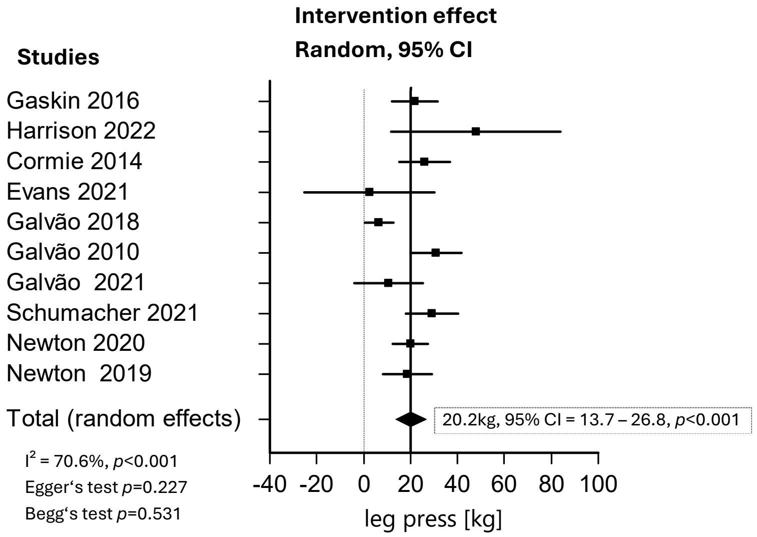

Figure S1 b

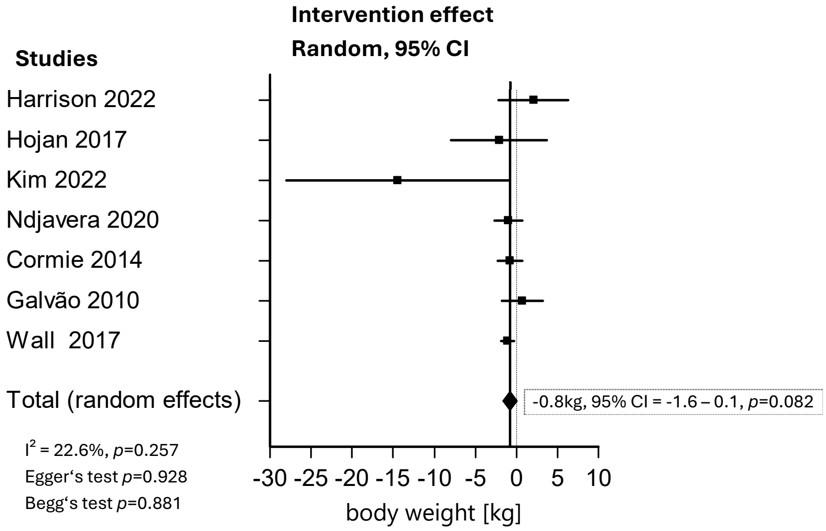

Figure S1 c

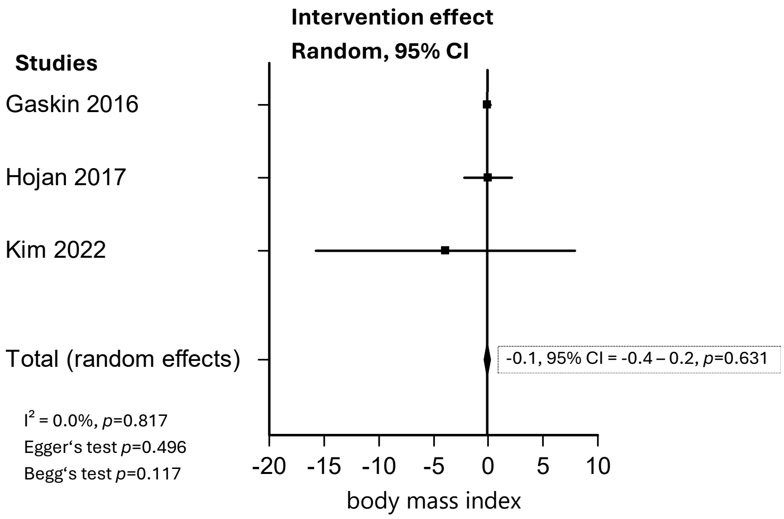

Figure S1 d

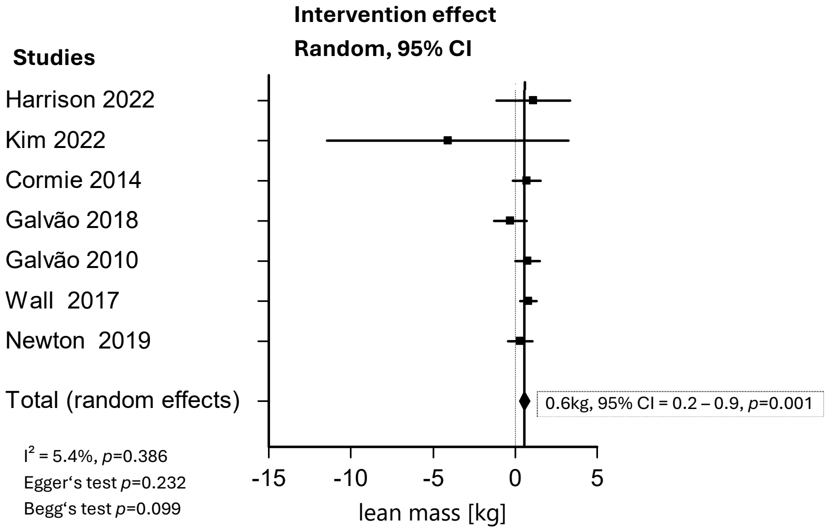

Figure S1 b

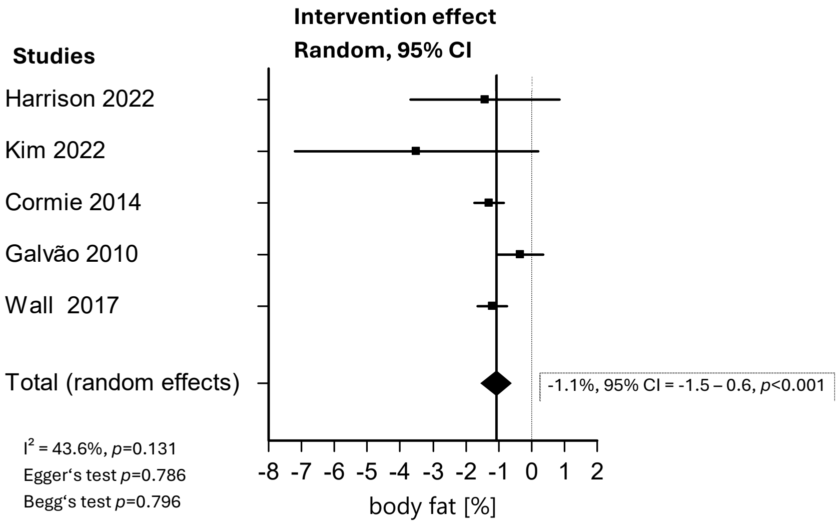

Figure S1 f

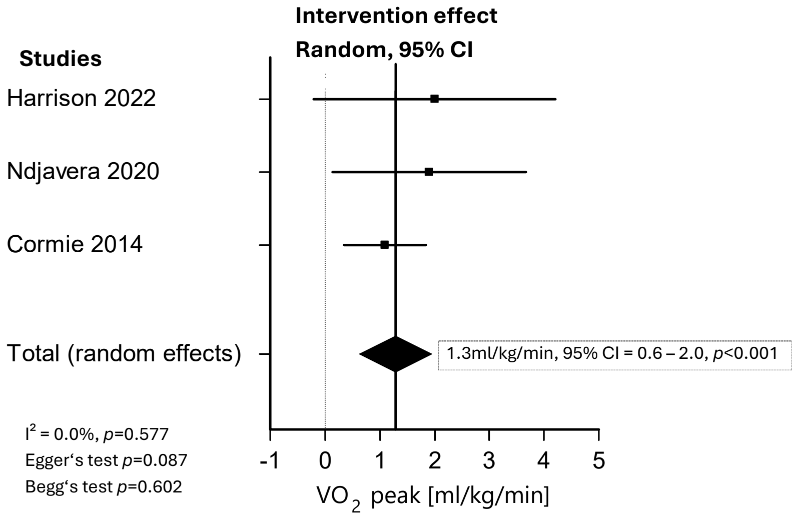

Figure S1 g

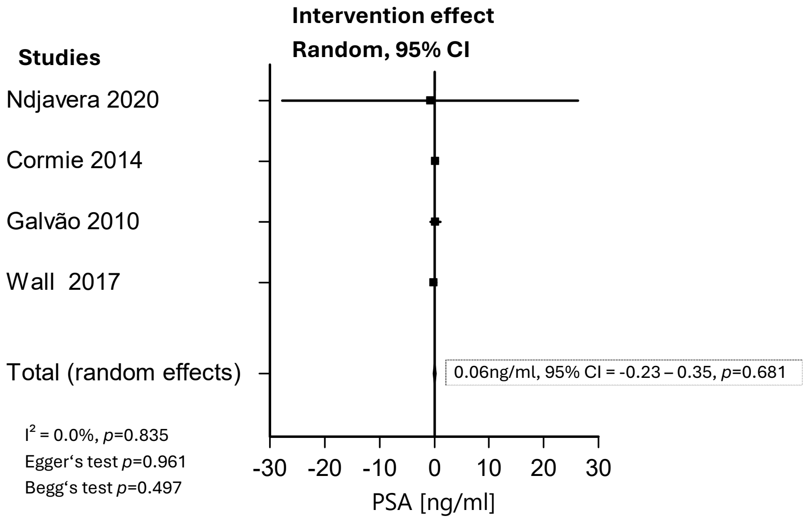

Figure S1 h

8. Forest Plots—Effects of Resistance Training Only (Figures S2 a–h)

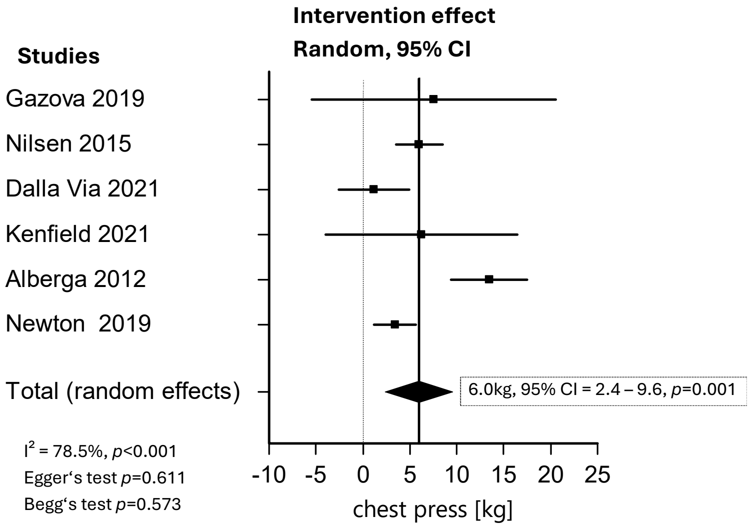

Figure S2 a

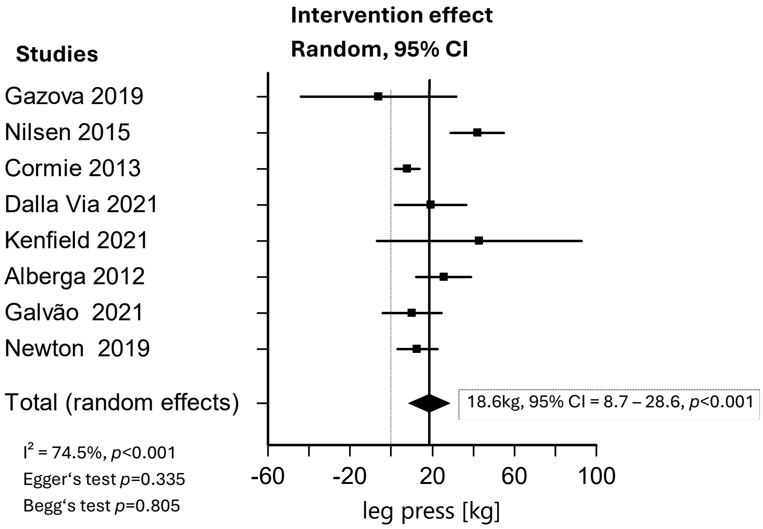

Figure S2 b

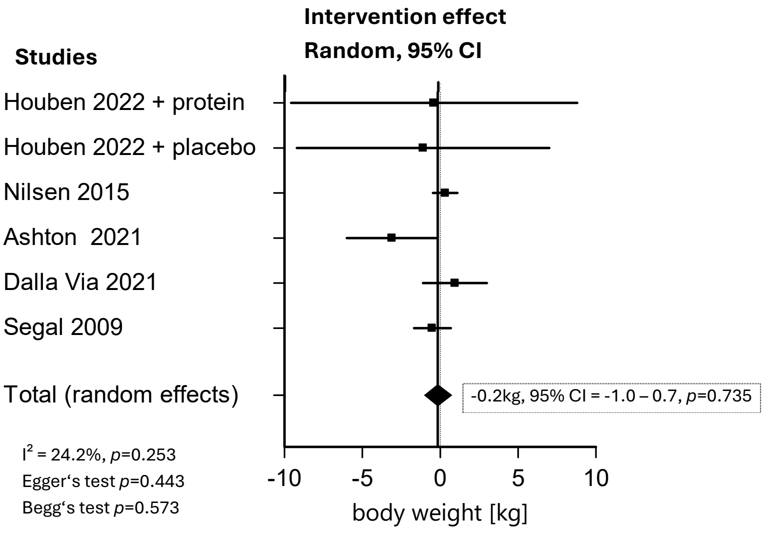

Figure S2 c

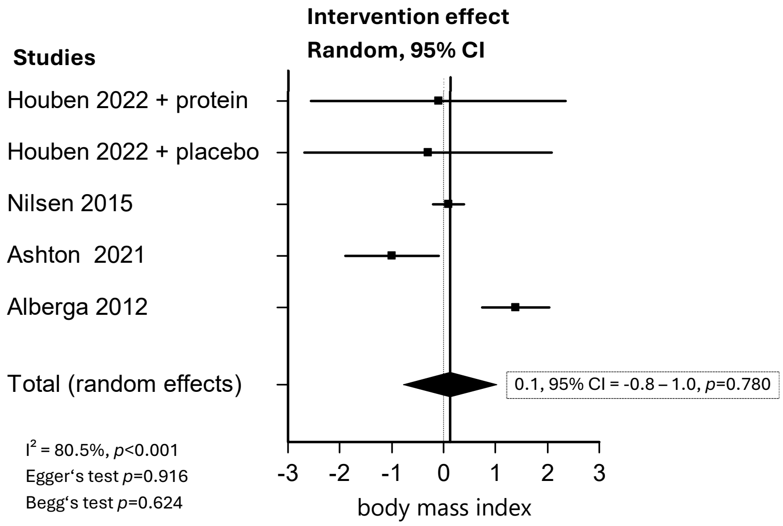

Figure S2 d

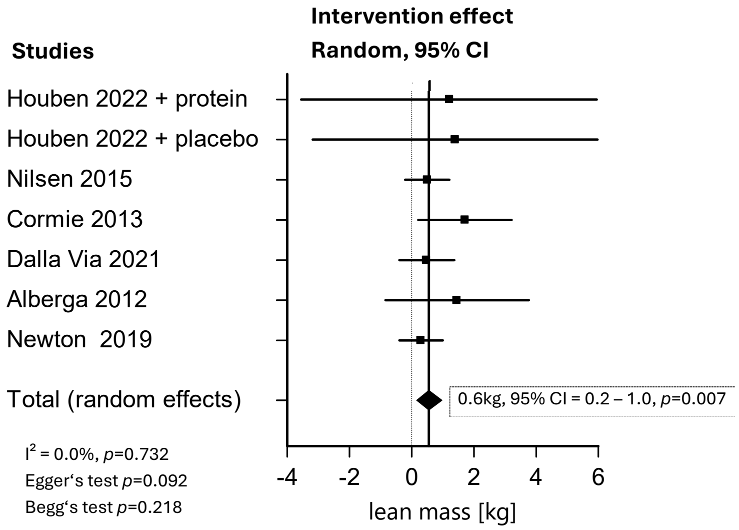

Figure S2 b

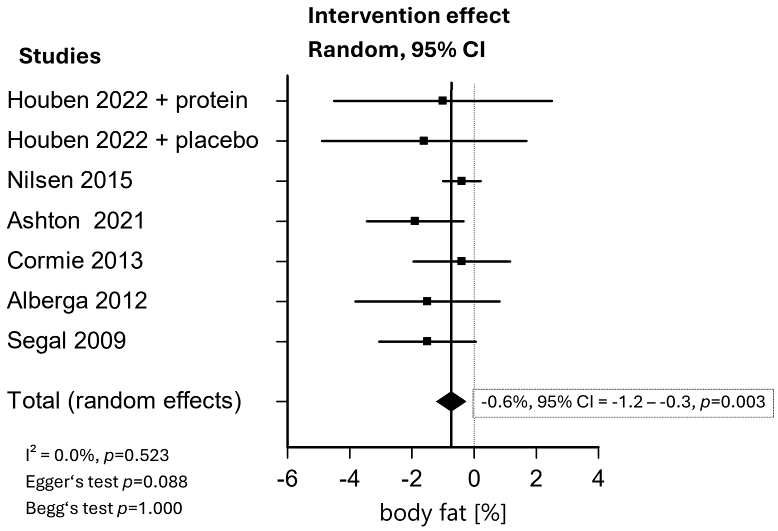

Figure S2 f

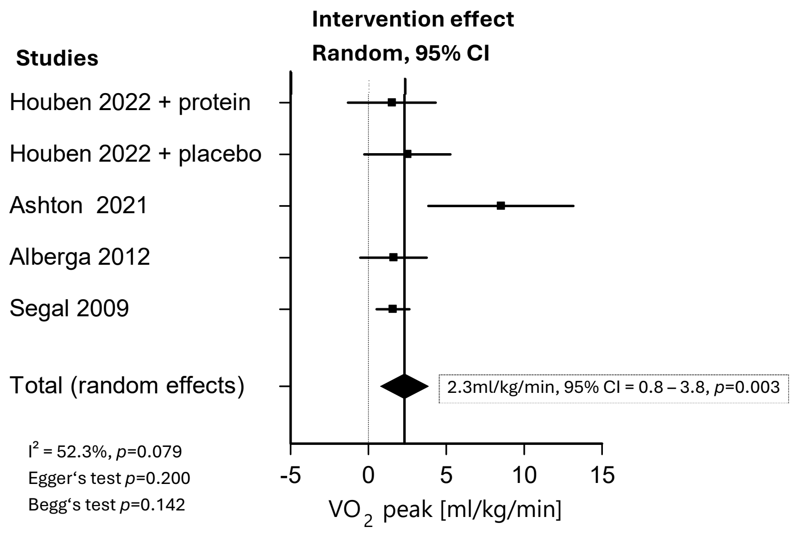

Figure S2 c

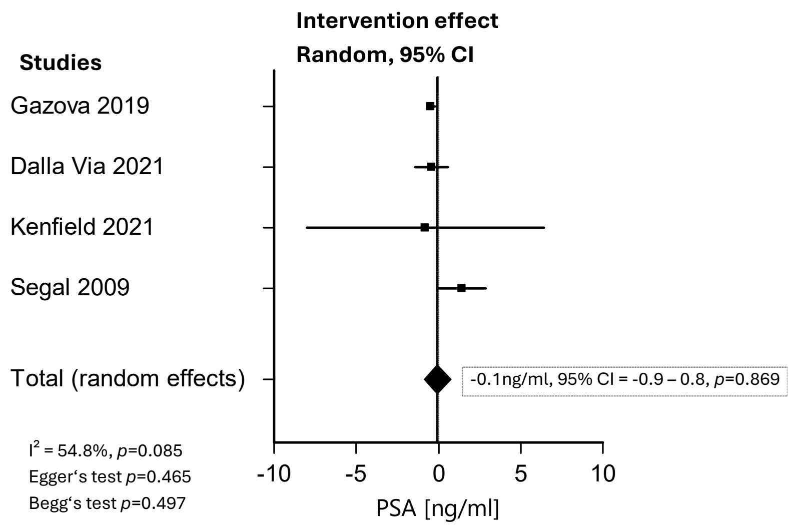

Figure S2 d

9. Funnel Plots—Effects of Combined Resistance and Aerobic Training (Figures S3 a–w)

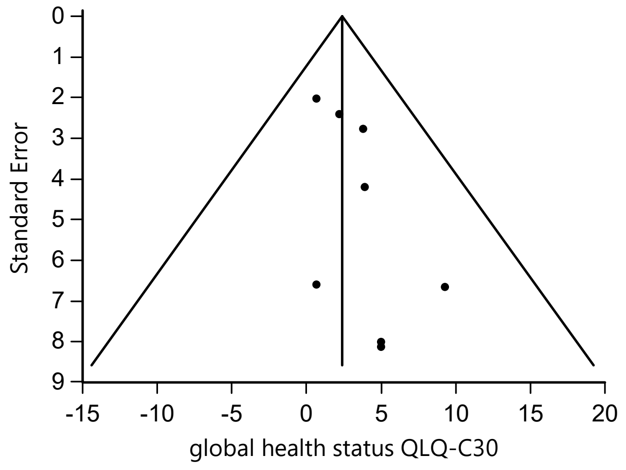

Figure S3 a

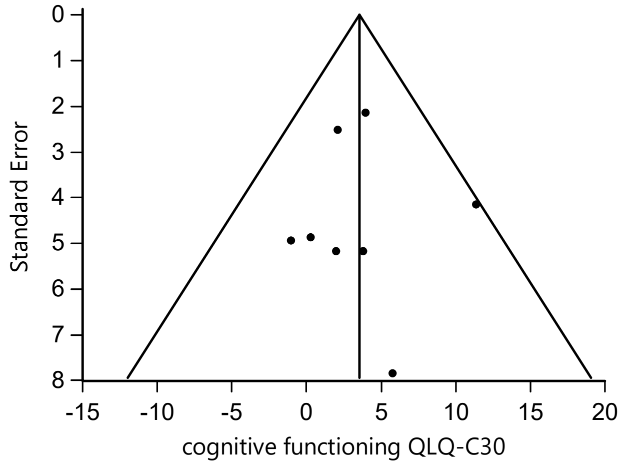

Figure S3 b

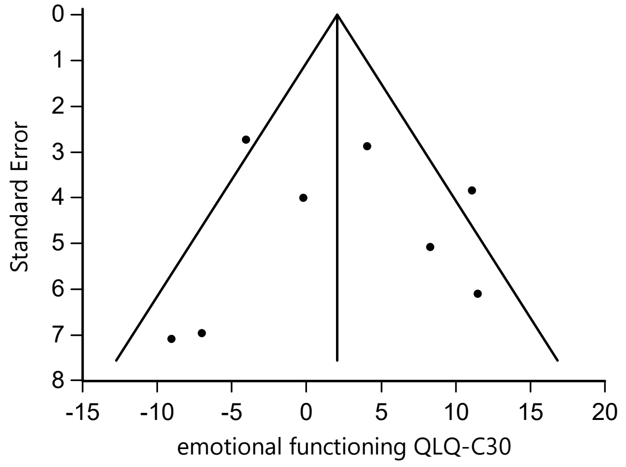

Figure S3 c

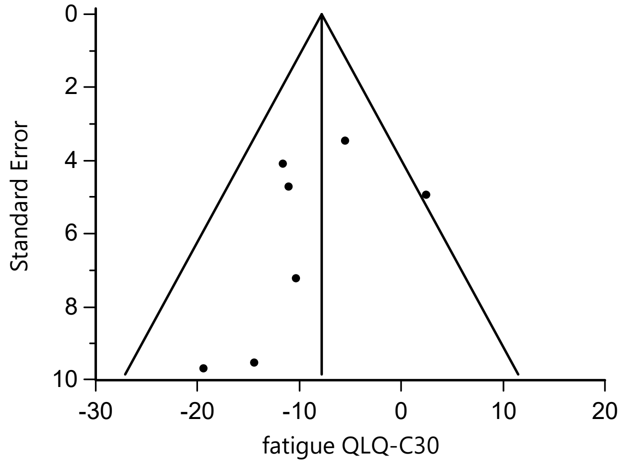

Figure S3 d

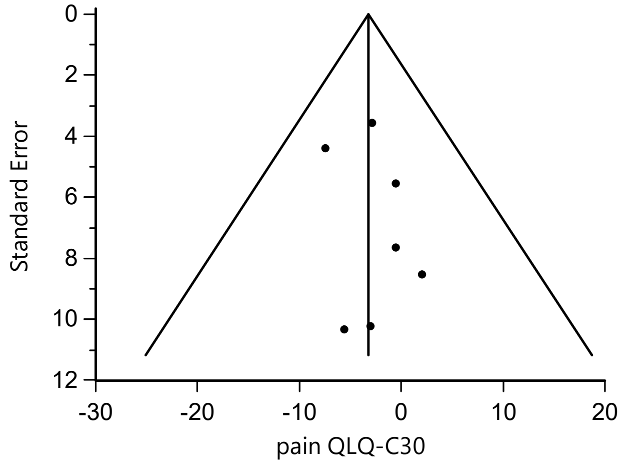

Figure S3 e

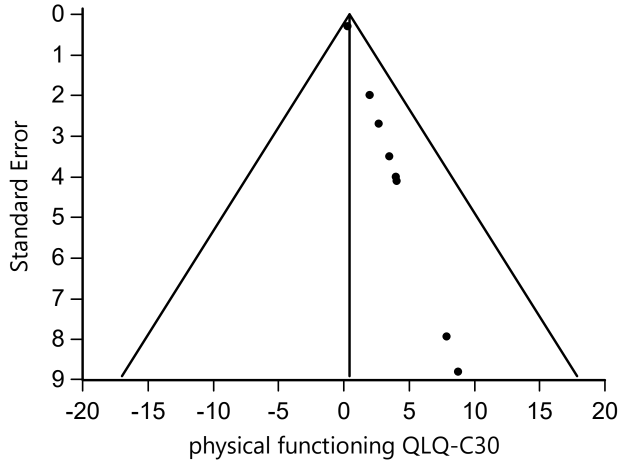

Figure S3 f

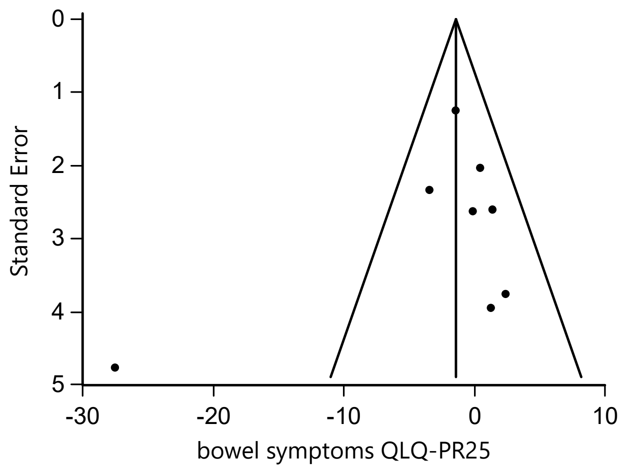

Figure S3 g

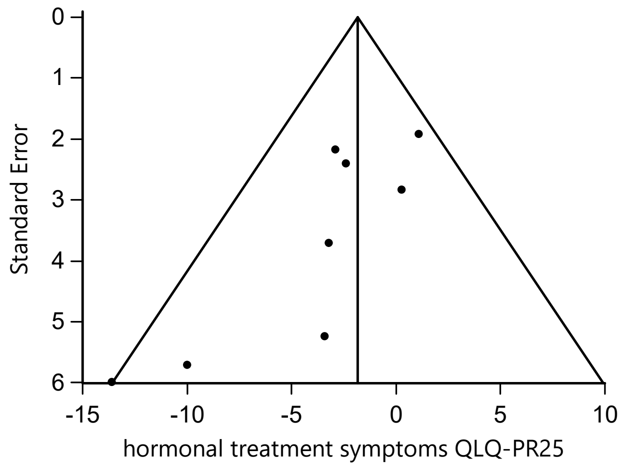

Figure S3 h

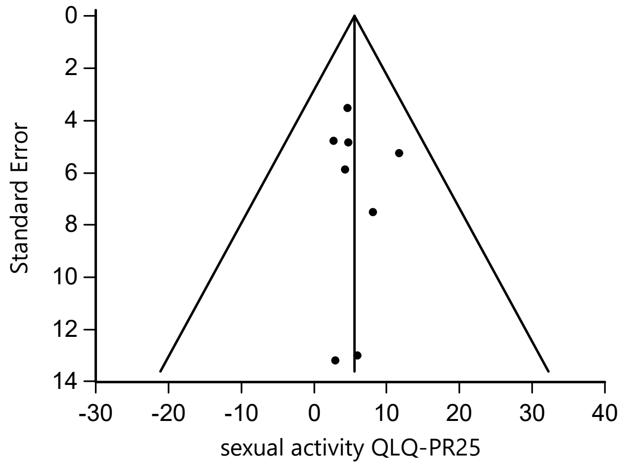

Figure S3 i

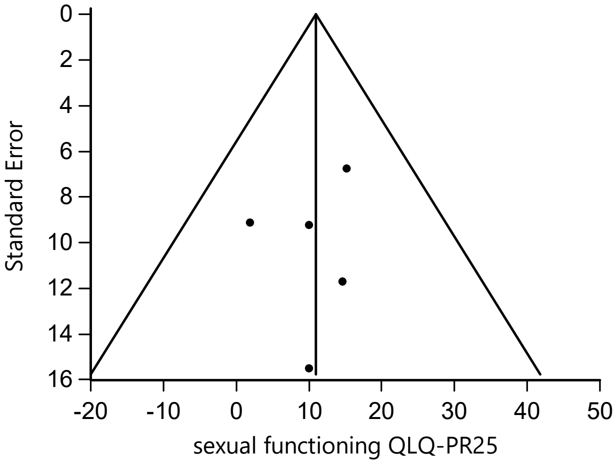

Figure S3 j

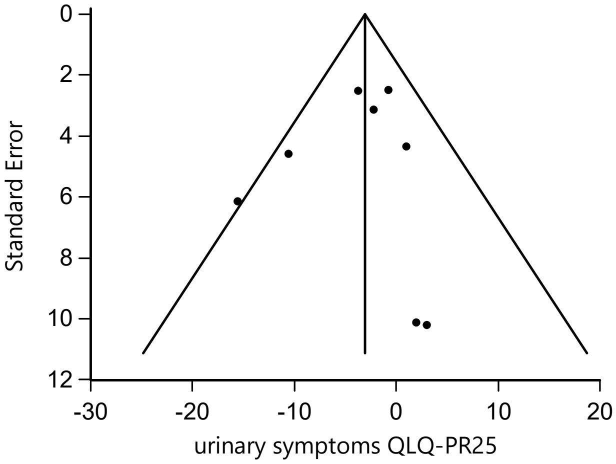

Figure S3 k

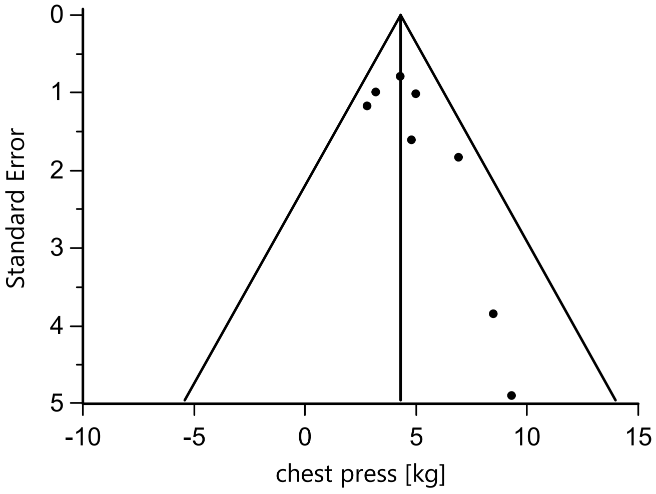

Figure S3 l

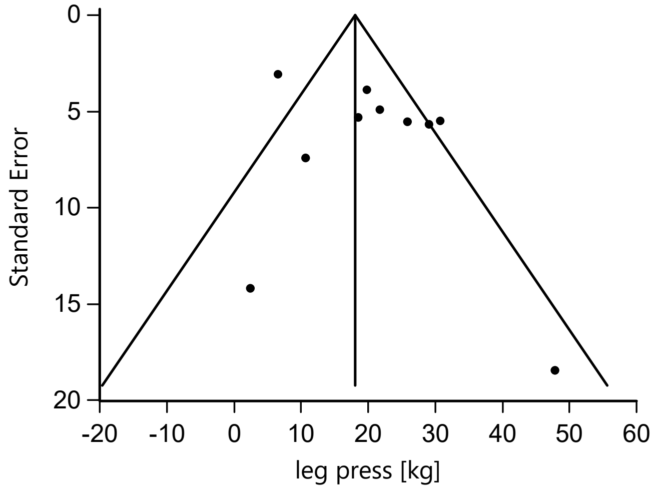

Figure S3 m

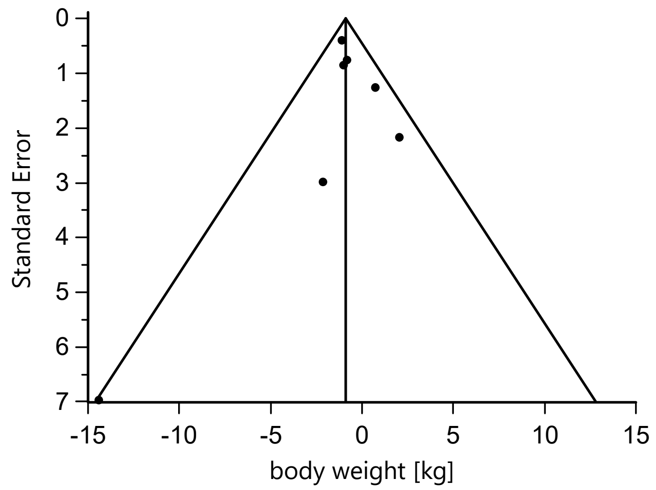

Figure S3 n

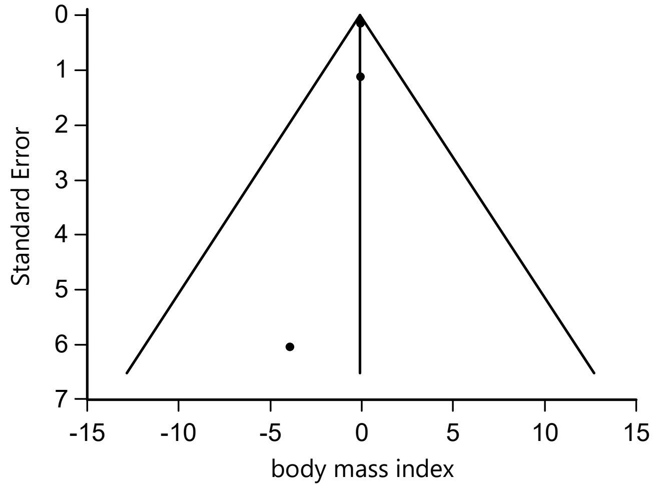

Figure S3 o

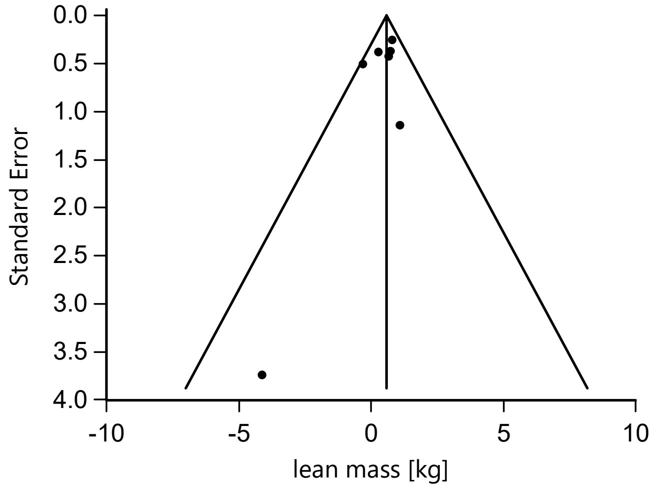

Figure S3 p

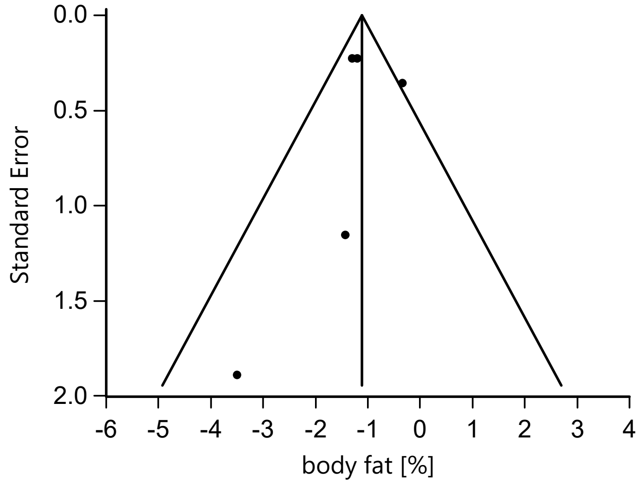

Figure S3 q

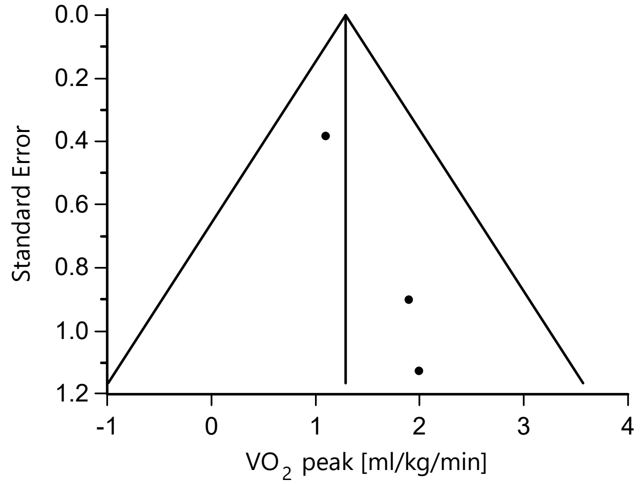

Figure S3 r

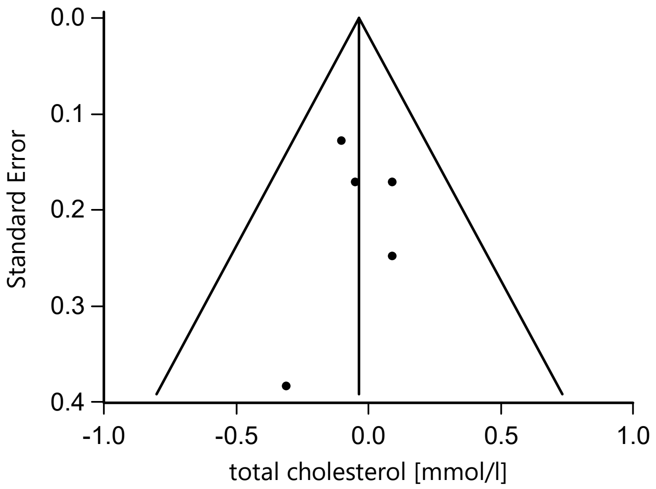

Figure S3 s

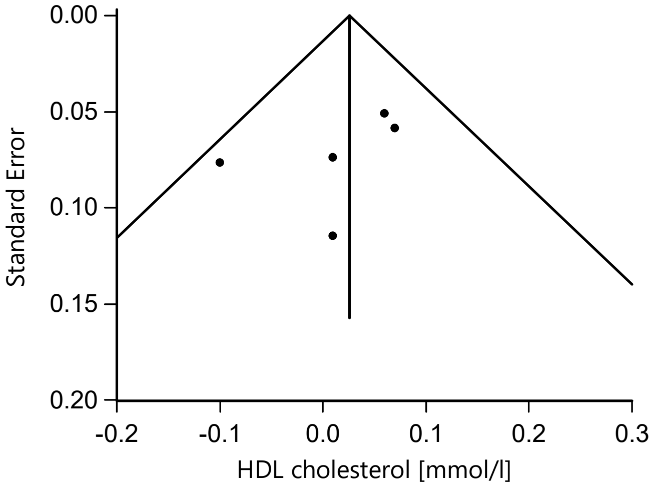

Figure S3 t

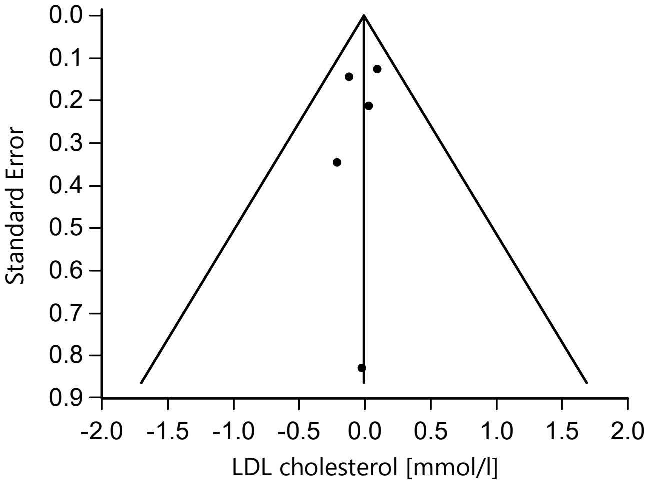

Figure S3 u

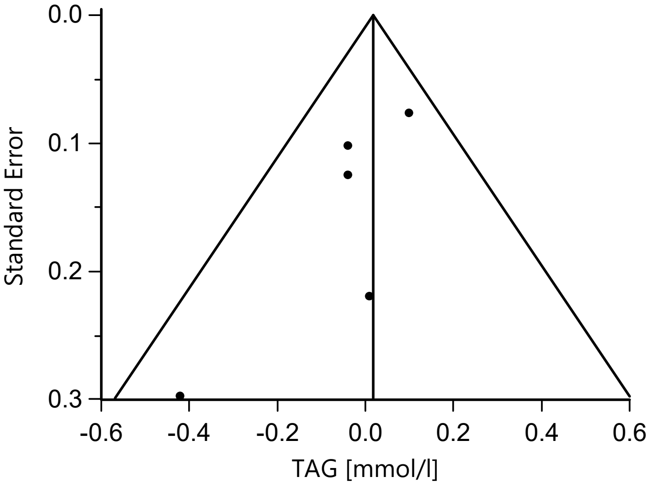

Figure S3 v

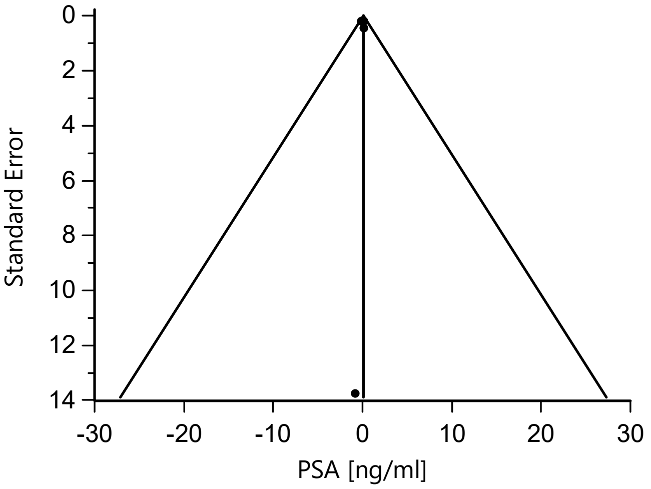

Figure S3 w

10. Funnel Plots—Effects of Resistance Training Only (Figures S4 a–h)

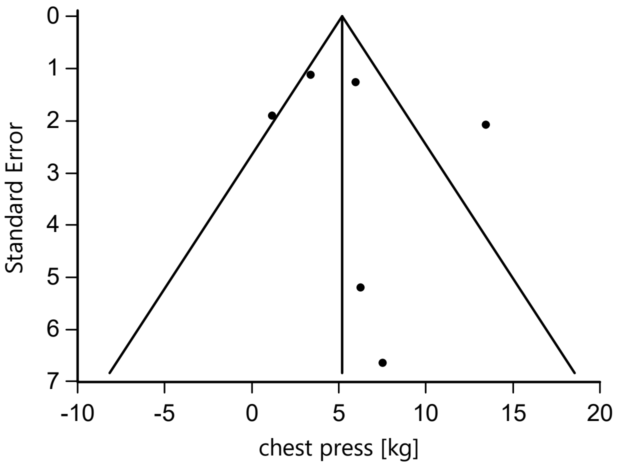

Figure S4 a

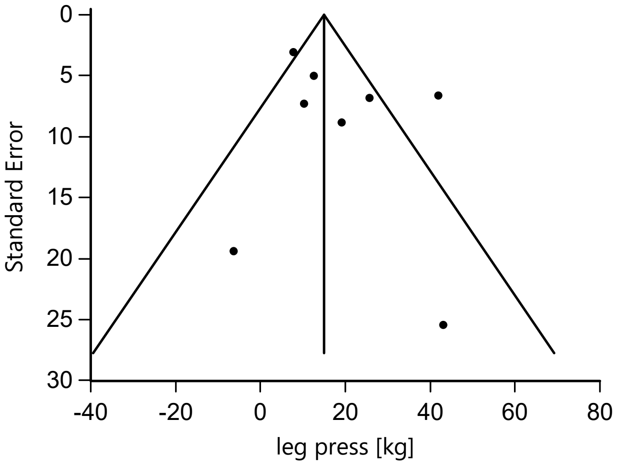

Figure S4 b

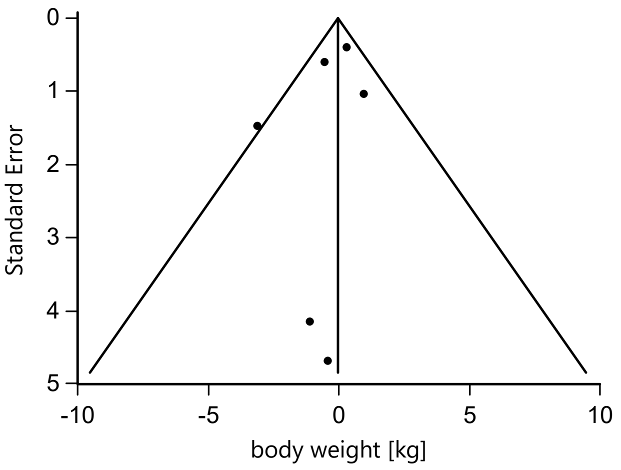

Figure S4 c

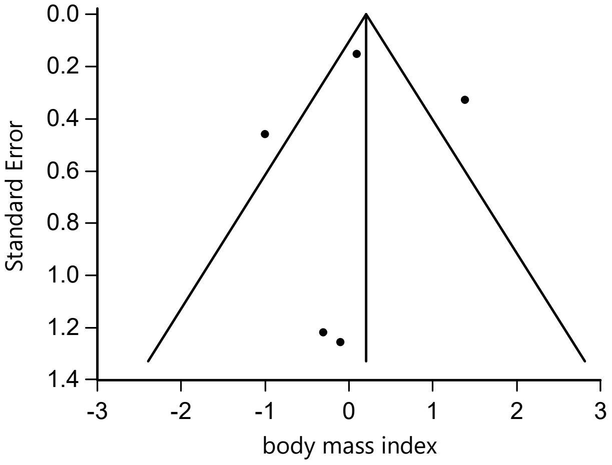

Figure S4 d

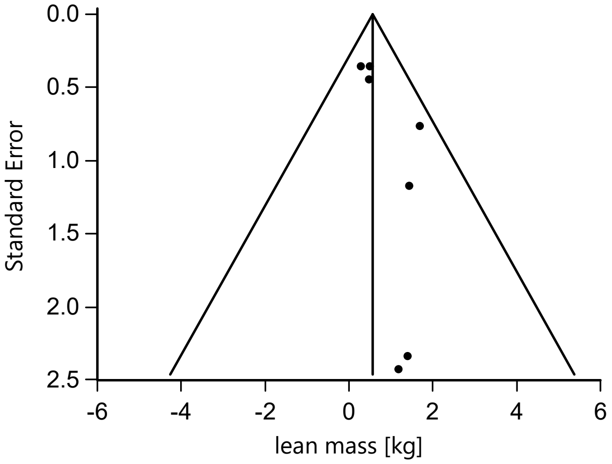

Figure S4 e

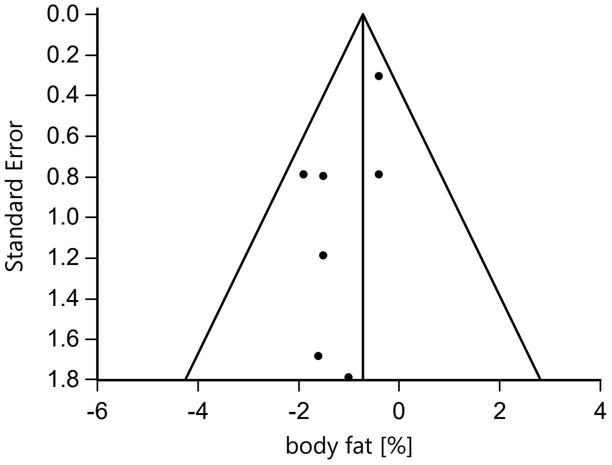

Figure S4 f

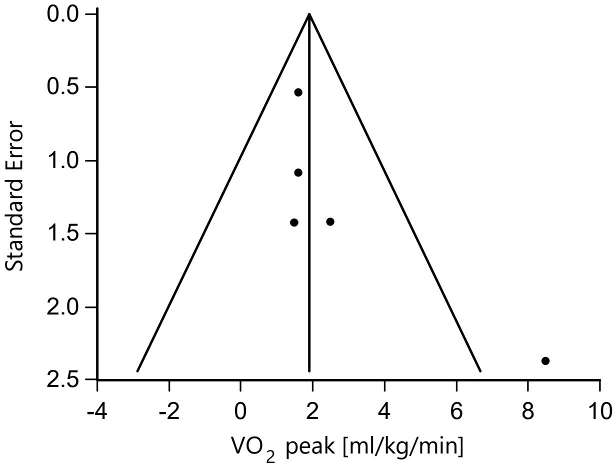

Figure S4 g

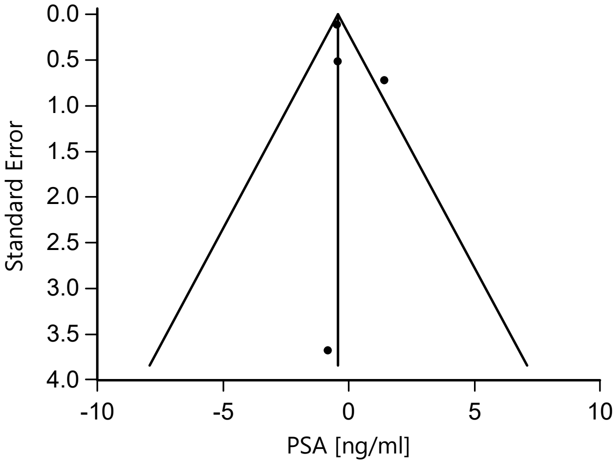

Figure S4 h

11. PRISMA 2020 for Abstracts Checklist (Table S4)

Table S4. PRISMA 2020 for Abstracts Checklist

Section and Topic	Item #	Checklist item	Reported (Yes/No)
**TITLE**			
Title	1	Identify the report as a systematic review.	Yes
**BACKGROUND**			
Objectives	2	Provide an explicit statement of the main objective(s) or question(s) the review addresses.	Yes
**METHODS**			
Eligibility criteria	3	Specify the inclusion and exclusion criteria for the review.	Yes
Information sources	4	Specify the information sources (e.g. databases, registers) used to identify studies and the date when each was last searched.	Yes
Risk of bias	5	Specify the methods used to assess risk of bias in the included studies.	Yes
Synthesis of results	6	Specify the methods used to present and synthesize results.	Yes
**RESULTS**			
Included studies	7	Give the total number of included studies and participants and summarize relevant characteristics of studies.	Yes
Synthesis of results	8	Present results for main outcomes, preferably indicating the number of included studies and participants for each. If meta-analysis was performed, report the summary estimate and confidence/credible interval. If comparing groups, indicate the direction of the effect (i.e. which group is favored).	Yes
**DISCUSSION**			
Limitations of evidence	9	Provide a brief summary of the limitations of the evidence included in the review (e.g. study risk of bias, inconsistency, and imprecision).	Yes
Interpretation	10	Provide a general interpretation of the results and important implications.	Yes
**OTHER**			
Funding	11	Specify the primary source of funding for the review.	No funding
Registration	12	Provide the register name and registration number.	Not in Abstract, but in Chapter 2.1

12. PRISMA 2020 Checklist (Table S5)

Table S5. PRISMA 2020 Checklist

Section and Topic	Item #	Checklist item	Location where item is reported
**TITLE**			
Title	1	Identify the report as a systematic review.	Title
**ABSTRACT**			
Abstract	2	See the PRISMA 2020 for Abstracts checklist.	Suppl. Table 3
**INTRODUCTION**			
Rationale	3	Describe the rationale for the review in the context of existing knowledge.	Chapter 1
Objectives	4	Provide an explicit statement of the objective(s) or question(s) the review addresses.	Chapter 1
**METHODS**			
Eligibility criteria	5	Specify the inclusion and exclusion criteria for the review and how studies were grouped for the syntheses.	Chapter 2.1
Information sources	6	Specify all databases, registers, websites, organizations, reference lists, and other sources searched or consulted to identify studies. Specify the date when each source was last searched or consulted.	Chapter 2.1
Search strategy	7	Present the full search strategies for all databases, registers, and websites, including any filters and limits used.	Chapter 2.1
Selection process	8	Specify the methods used to decide whether a study met the inclusion criteria of the review, including how many reviewers screened each record and each report retrieved, whether they worked independently and, if applicable, details of automation tools used in the process.	Chapter 2.1
Data collection process	9	Specify the methods used to collect data from reports, including how many reviewers collected data from each report, whether they worked independently, any processes for obtaining or confirming data from study investigators and, if applicable, details of automation tools used in the process.	Chapter 2.2
Data items	10a	List and define all outcomes for which data were sought. Specify whether all results that were compatible with each outcome domain in each study were sought (e.g. for all measures, time points, analyses) and, if not, the methods used to decide which results to collect.	Chapter 2.2
	10b	List and define all other variables for which data were sought (e.g. participant and intervention characteristics, funding sources). Describe any assumptions made about any missing or unclear information.	Chapter 2.2
Study risk of bias assessment	11	Specify the methods used to assess risk of bias in the included studies, including details of the tool(s) used, how many reviewers assessed each study, whether they worked independently and, if applicable, details of automation tools used in the process.	Chapter 2.4
Effect measures	12	Specify for each outcome the effect measure(s) (e.g. risk ratio, mean difference) used in the synthesis or presentation of results.	Chapter 2.4

Section and Topic	Item #	Checklist item	Location where item is reported
Synthesis methods	13a	Describe the processes used to decide which studies were eligible for each synthesis (e.g. tabulating the study intervention characteristics and comparing against the planned groups for each synthesis (item #5)).	Chapter 2.1
	13b	Describe any methods required to prepare the data for presentation or synthesis, such as handling of missing summary statistics, or data conversions.	Chapter 2.4
	13c	Describe any methods used to tabulate or visually display results of individual studies and syntheses.	Chapter 2.4
	13d	Describe any methods used to synthesize results and provide a rationale for the choice(s). If meta-analysis was performed, describe the model(s), method(s) to identify the presence and extent of statistical heterogeneity and software package(s) used.	Chapter 2.4
	13e	Describe any methods used to explore possible causes of heterogeneity among study results (e.g. subgroup analysis, meta-regression).	Chapter 2.3
	13f	Describe any sensitivity analyses conducted to assess robustness of the synthesized results.	Not conducted
Reporting bias assessment	14	Describe any methods used to assess risk of bias due to missing results in a synthesis (arising from reporting biases).	Chapter 2.4
Certainty assessment	15	Describe any methods used to assess certainty (or confidence) in the body of evidence for an outcome.	Chapter 2.3
**RESULTS**			
Study selection	16a	Describe the results of the search and selection process, from the number of records identified in the search to the number of studies included in the review, ideally using a flow diagram.	Chapter 3.1, Figure 1
	16b	Cite studies that might appear to meet the inclusion criteria, but which were excluded, and explain why they were excluded.	Chapter 3.1
Study characteristics	17	Cite each included study and present its characteristics.	Table 1
Risk of bias in studies	18	Present assessments of risk of bias for each included study.	Suppl. Table 2
Results of individual studies	19	For all outcomes, present, for each study: (a) summary statistics for each group (where appropriate) and (b) an effect estimate and its precision (e.g. confidence/credible interval), ideally using structured tables or plots.	Figure 2
Results of syntheses	20a	For each synthesis, briefly summarize the characteristics and risk of bias among contributing studies.	Chapter 3.3–3.6
	20b	Present results of all statistical syntheses conducted. If meta-analysis was performed, present for each the summary estimate and its precision (e.g. confidence/credible interval) and measures of statistical heterogeneity. If comparing groups, describe the direction of the effect.	Chapter 3.3–3.6
	20c	Present results of all investigations of possible causes of heterogeneity among study results.	Chapter 3.7
	20d	Present results of all sensitivity analyses conducted to assess the robustness of the synthesized results.	Not conducted
Reporting biases	21	Present assessments of risk of bias due to missing results (arising from reporting biases) for each synthesis assessed.	Chapter 3.7

Section and Topic	Item #	Checklist item	Location where item is reported
Certainty of evidence	22	Present assessments of certainty (or confidence) in the body of evidence for each outcome assessed.	Suppl. Table 2
**DISCUSSION**			
Discussion	23a	Provide a general interpretation of the results in the context of other evidence.	Chapter 4
	23b	Discuss any limitations of the evidence included in the review.	Chapter 4
	23c	Discuss any limitations of the review processes used.	Chapter 4
	23d	Discuss implications of the results for practice, policy, and future research.	Chapter 4
**OTHER INFORMATION**			
Registration and protocol	24a	Provide registration information for the review, including register name and registration number, or state that the review was not registered.	Chapter 2.1, Suppl. Chapter 2
	24b	Indicate where the review protocol can be accessed, or state that a protocol was not prepared.	Not prepared
	24c	Describe and explain any amendments to information provided at registration or in the protocol.	No amendments
Support	25	Describe sources of financial or non-financial support for the review, and the role of the funders or sponsors in the review.	The authors received no financial support for the research, authorship and/or publication of this article.
Competing interests	26	Declare any competing interests of review authors.	No competing interests have to be declared.
Availability of data, code and other materials	27	Report which of the following are publicly available and where they can be found: template data collection forms; data extracted from included studies; data used for all analyses; analytic code; any other materials used in the review.	Upon request from the corresponding author

References

1. Livingston PM, Craike MJ, Salmon J, et al. Effects of a Clinician Referral and Exercise Program for Men Who Have Completed Active Treatment for Prostate Cancer: A Multicenter Cluster Randomized Controlled Trial (ENGAGE). *Cancer*. 2015;121(15):2646. doi:10.1002/CNCR.29385
2. Gaskin CJ, Fraser SF, Owen PJ, Craike M, Orellana L, Livingston PM. Fitness outcomes from a randomised controlled trial of exercise training for men with prostate cancer: the ENGAGE study. *J Cancer Surviv*. 2016;10(6):972-980. doi:10.1007/S11764-016-0543-6
3. Harrison MR, Davis PG, Khouri MG, et al. A randomized controlled trial comparing changes in fitness with or without supervised exercise in patients initiated on enzalutamide and androgen deprivation therapy for non-metastatic castration-sensitive prostate cancer (EXTEND). *Prostate Cancer Prostatic Dis*. 2022;25(1):58-64. doi:10.1038/S41391-022-00519-4
4. Hojan K, Kwiatkowska-Borowczyk E, Leporowska E, Milecki P. Inflammation, cardiometabolic markers, and functional changes in men with prostate cancer. A randomized controlled trial of a 12-month exercise program. *Pol Arch Intern Med*. 2017;127(1):25-35. doi:10.20452/PAMW.3888
5. Kim JS, Taaffe DR, Galvão DA, et al. Exercise in advanced prostate cancer elevates myokine levels and suppresses in-vitro cell growth. *Prostate Cancer Prostatic Dis*. 2022;25(1):86. doi:10.1038/S41391-022-00504-X
6. Mardani A, Pedram Razi S, Mazaheri R, Haghani S, Vaismoradi M. Effect of the exercise programme on the quality of life of prostate cancer survivors: A randomized controlled trial. *Int J Nurs Pract*. 2021;27(2). doi:10.1111/IJN.12883
7. Ndjavera W, Orange ST, O'Doherty AF, et al. Exercise-induced attenuation of treatment side-effects in patients with newly diagnosed prostate cancer beginning androgen-deprivation therapy: a randomised controlled trial. *BJU Int*. 2020;125(1):28-37. doi:10.1111/BJU.14922
8. Ax AK, Johansson B, Lyth J, Nordin K, Börjeson S. Short- and long-term effect of high versus low-to-moderate intensity exercise to optimise health-related quality of life after oncological treatment—results from the Phys-Can project. *Supportive Care in Cancer*. 2022;30(7):5949-5963. https://doi.org/10.1007/S00520-022-07016-3
9. Cormie P, Galvão DA, Spry N, et al. Can supervised exercise prevent treatment toxicity in patients with prostate cancer initiating androgen-deprivation therapy: a randomised controlled trial. *BJU Int*. 2015;115(2):256-266. doi:10.1111/bju.12646
10. Cormie P, Newton RU, Taaffe DR, et al. Exercise maintains sexual activity in men undergoing androgen suppression for prostate cancer: a randomized controlled trial. *Prostate Cancer Prostatic Dis*. 2013;16(2):170-175. doi:10.1038/PCAN.2012.52

11. Evans HEL, Galvão DA, Forbes CC, et al. Acceptability and Preliminary Efficacy of a Web- and Telephone-Based Personalised Exercise Intervention for Individuals with Metastatic Prostate Cancer: The ExerciseGuide Pilot Randomised Controlled Trial. *Cancers (Basel)*. 2021;13(23). doi:10.3390/CANCERS13235925
12. Galvão DA, Taaffe DR, Spry N, et al. Exercise Preserves Physical Function in Prostate Cancer Patients with Bone Metastases. *Med Sci Sports Exerc*. 2018;50(3):393. doi:10.1249/MSS.0000000000001454
13. Galvão DA, Taaffe DR, Spry N, Joseph D, Newton RU. Combined resistance and aerobic exercise program reverses muscle loss in men undergoing androgen suppression therapy for prostate cancer without bone metastases: a randomized controlled trial. *J Clin Oncol*. 2010;28(2):340-347. doi:10.1200/JCO.2009.23.2488
14. Galvão DA, Newton RU, Chambers SK, et al. Psychological distress in men with prostate cancer undertaking androgen deprivation therapy: modifying effects of exercise from a year-long randomized controlled trial. *Prostate Cancer and Prostatic Diseases 2021 24:3*. 2021;24(3):758-766. doi:10.1038/s41391-021-00327-2
15. Galvão DA, Taaffe DR, Chambers SK, et al. Exercise intervention and sexual function in advanced prostate cancer: a randomised controlled trial. *BMJ Support Palliat Care*. 2022;12(1):29-32. doi:10.1136/BMJSPCARE-2020-002706
16. Schumacher O, Galvão DA, Taaffe DR, et al. Effect of Exercise Adjunct to Radiation and Androgen Deprivation Therapy on Patient-Reported Treatment Toxicity in Men With Prostate Cancer: A Secondary Analysis of 2 Randomized Controlled Trials. *Pract Radiat Oncol*. 2021;11(3):215-225. doi:10.1016/j.prro.2021.01.005
17. Wall BA, Galvão DA, Fatehee N, et al. Exercise improves VO2max and body composition in androgen deprivation therapy-treated prostate cancer patients. *Med Sci Sports Exerc*. 2017;49(8):1503-1510. doi:10.1249/MSS.0000000000001277
18. Newton RU, Galvão DA, Spry N, et al. Timing of exercise for muscle strength and physical function in men initiating ADT for prostate cancer. *Prostate Cancer Prostatic Dis*. 2020;23(3):457-464. doi:10.1038/S41391-019-0200-Z
19. Newton RU, Galvão DA, Spry N, et al. Exercise Mode Specificity for Preserving Spine and Hip Bone Mineral Density in Prostate Cancer Patients. *Med Sci Sports Exerc*. 2019;51(4):607-614. doi:10.1249/MSS.0000000000001831
20. Gazova A, Samakova A, Laczo E, et al. Clinical utility of miRNA-1, miRNA-29g and miRNA-133s plasma levels in prostate cancer patients with high-intensity training after androgen-deprivation therapy. *Physiol Res*. 2019;68(Suppl 2):S139-S147. doi:10.33549/PHYSIOLRES.934298
21. Houben LHP, Overkamp M, Van Kraaij P, et al. Resistance Exercise Training Increases Muscle Mass and Strength in Prostate Cancer Patients on Androgen

Deprivation Therapy. *Med Sci Sports Exerc*. 2023;55(4):614-624. doi:10.1249/MSS.0000000000003095
22. Nilsen TS, Raastad T, Skovlund E, et al. Effects of strength training on body composition, physical functioning, and quality of life in prostate cancer patients during androgen deprivation therapy. *Acta Oncol (Madr)*. 2015;54(10):1805-1813. doi:10.3109/0284186X.2015.1037008
23. Ashton RE, Aning JJ, Tew GA, Robson WA, Saxton JM. Supported progressive resistance exercise training to counter the adverse side effects of robot-assisted radical prostatectomy: a randomised controlled trial. *Support Care Cancer*. 2021;29(8):4595-4605. doi:10.1007/S00520-021-06002-5
24. Cormie P, Newton RU, Spry N, Joseph D, Taaffe DR, Galvão DA. Safety and efficacy of resistance exercise in prostate cancer patients with bone metastases. *Prostate Cancer Prostatic Dis*. 2013;16(4):328-335. doi:10.1038/PCAN.2013.22
25. Dalla Via J, Owen PJ, Daly RM, et al. Musculoskeletal Responses to Exercise Plus Nutrition in Men with Prostate Cancer on Androgen Deprivation: A 12-Month RCT. *Med Sci Sports Exerc*. 2021;53(10):2054-2065. doi:10.1249/MSS.0000000000002682
26. Langlais CS, Chen YH, Van Blarigan EL, et al. Quality of life for men with metastatic castrate-resistant prostate cancer participating in an aerobic and resistance exercise pilot intervention. *Urol Oncol*. 2023;41(3):146.e1-146.e11. doi:10.1016/J.UROLONC.2022.11.016
27. Kenfield SA, Van Blarigan EL, Panchal N, et al. Feasibility, safety, and acceptability of a remotely monitored exercise pilot CHAMP: A Clinical trial of High-intensity Aerobic and resistance exercise for Metastatic castrate-resistant Prostate cancer. *Cancer Med*. 2021;10(22):8058-8070. doi:10.1002/CAM4.4324
28. Alberga AS, Segal RJ, Reid RD, et al. Age and androgen-deprivation therapy on exercise outcomes in men with prostate cancer. *Supportive Care in Cancer*. 2012;20(5):971-981. doi:10.1007/S00520-011-1169-X
29. Segal RJ, Reid RD, Courneya KS, et al. Randomized Controlled Trial of Resistance or Aerobic Exercise in Men Receiving Radiation Therapy for Prostate Cancer. *Journal of Clinical Oncology*. 2009;27(3):344-351. doi:10.1200/JCO.2007.15.4963
30. Sheill G, Brady L, Hayes B, et al. ExPeCT: a randomised trial examining the impact of exercise on quality of life in men with metastatic prostate cancer. *Supportive Care in Cancer*. 2023;31(5):1-11. doi:10.1007/S00520-023-07740-4
